# Spatiotemporal Pattern of Ectopic Cell Divisions Contribute to Mis-Shaped Phenotype of Primary and Lateral Roots of *katanin1* Mutant

**DOI:** 10.3389/fpls.2020.00734

**Published:** 2020-06-09

**Authors:** Miroslav Ovečka, Ivan Luptovčiak, George Komis, Olga Šamajová, Despina Samakovli, Jozef Šamaj

**Affiliations:** Department of Cell Biology, Centre of the Region Haná for Biotechnological and Agricultural Research, Faculty of Science, Palacký University Olomouc, Olomouc, Czechia

**Keywords:** *Arabidopsis*, ectopic cell division, light-sheet fluorescence microscopy, live cell imaging, katanin, microtubules, root development

## Abstract

Pattern formation, cell proliferation, and directional cell growth, are driving factors of plant organ shape, size, and overall vegetative development. The establishment of vegetative morphogenesis strongly depends on spatiotemporal control and synchronization of formative and proliferative cell division patterns. In this context, the progression of cell division and the regulation of cell division plane orientation are defined by molecular mechanisms converging to the proper positioning and temporal reorganization of microtubule arrays such as the preprophase microtubule band, the mitotic spindle and the cytokinetic phragmoplast. By focusing on the tractable example of primary root development and lateral root emergence in *Arabidopsis thaliana*, genetic studies have highlighted the importance of mechanisms underlying microtubule reorganization in the establishment of the root system. In this regard, severe alterations of root growth, and development found in extensively studied *katanin1* mutants of *A. thaliana* (*fra2, lue1*, and *ktn1-2*), were previously attributed to defective rearrangements of cortical microtubules and aberrant cell division plane reorientation. How KATANIN1-mediated microtubule severing contributes to tissue patterning and organ morphogenesis, ultimately leading to anisotropy in microtubule organization is a trending topic under vigorous investigation. Here we addressed this issue during root development, using advanced light-sheet fluorescence microscopy (LSFM) and long-term imaging of *ktn1-2* mutant expressing the GFP-TUA6 microtubule marker. This method allowed spatial and temporal monitoring of cell division patterns in growing roots. Analysis of acquired multidimensional data sets revealed the occurrence of ectopic cell divisions in various tissues including the calyptrogen and the protoxylem of the main root, as well as in lateral root primordia. Notably the *ktn1-2* mutant exhibited excessive longitudinal cell divisions (parallel to the root axis) at ectopic positions. This suggested that changes in the cell division pattern and the occurrence of ectopic cell divisions contributed significantly to pleiotropic root phenotypes of *ktn1-2* mutant. LSFM provided evidence that KATANIN1 is required for the spatiotemporal control of cell divisions and establishment of tissue patterns in living *A. thaliana* roots.

## Introduction

Plant roots are regularly used for the microscopical characterization of plant body organization and pattern formation in various plant developmental studies. As exemplified by extensive studies in the model plant *Arabidopsis thaliana*, all tissues of the root apical meristem are derived from the activity of cells organized in the stem cell niche (Dolan et al., 1993; Parizot et al., 2008). Importantly, root pattern formation and cell fate specification within the root meristem are established by asymmetric formative cell divisions within the stem cell niche. Discrete cell lineages produced by formative cell divisions are arranged in concentric layers and further propagate along the root axis, via symmetric proliferative anticlinal divisions with transverse orientation of the cell division plane (CDP) to the root axis (Van Norman, 2016).

Tissue patterning and organ formation in plants are hallmarked by the orientation of CDP (Rasmussen et al., 2011; Müller, 2012) and cell growth directionality (Deinum and Mulder, 2013). CDP is predicted by the assembly of a cortical microtubule annulus, the preprophase microtubule band (PPB; Rasmussen et al., 2011; Smertenko et al., 2017), while cell growth directionality is mainly mediated by specific arrangement of cortical microtubules intimately associated with the likewise oriented cellulose deposition in the overlying cell wall (Komis et al., 2014; Polko and Kieber, 2019 and references therein).

Demarcation of CDP orientation by the PPB long after its disassembly at nuclear envelope breakdown during prophase (Rasmussen et al., 2011; Smertenko et al., 2017) is thought to be mediated by the persistent recruitment of molecular markers at the site where the daughter cell wall will fuse with the parent cell wall during cytokinesis (Livanos and Müller, 2019). Proteins which occupy the cell division zone long after the disassembly of the PPB (Smertenko et al., 2017), include the FASS/TONNEAU protein phosphatase 2A subunit (Spinner et al., 2013), the Ran GTPase Activating Protein 1 (RanGAP1; Xu et al., 2008), the Phragmoplast Orienting Kinesins 1 and 2 (POK1/2; Müller et al., 2006), the protein TANGLED (Walker et al., 2007; Rasmussen et al., 2011), the protein AIR9 (Buschmann et al., 2006) and the microtubule crosslinking protein MAP65-4 (Li et al., 2017), which form thin cortical rings coinciding with the PPB and the future sites of cell plate insertion. Importantly, signaling molecules like the mitogen activated protein kinases MPK6 and MPK4 had been localized to the PPB and the phragmoplast, suggesting that both CDP determination and cytokinetic progression might be regulated by phosphorylation of cytoskeletal substrates, like members of the MAP65 family of microtubule crosslinkers (Beck et al., 2010, 2011; Kosetsu et al., 2010; Müller et al., 2010; Smékalová et al., 2014). Mutants of the above proteins exhibit aberrant CDP orientation (Camilleri et al., 2002; Xu et al., 2008; Müller et al., 2010; Smékalová et al., 2014).

During plant growth many developmental events depend on the precise control of CDP orientation. Embryo, root meristem, stomata, and the male germline formation in plants are characteristic plant developmental processes governed by asymmetric cell divisions (Metzinger and Bergmann, 2010). Both symmetric and asymmetric cell division are generally predicted by the respective symmetric or asymmetric positioning of the PPB. The process of PPB formation, involves the rearrangement of the cortical microtubule network to a progressively narrowing cortical microtubule ring (Komis et al., 2017; Smertenko et al., 2017). This process is supported and controlled by different microtubule-associated proteins (Hamada, 2014).

*KATANIN1*, encodes for the catalytic p60 subunit of a microtubule severing complex, and plays central roles in mechanisms governing microtubule dynamic reorganization in plants (Lindeboom et al., 2013; Luptovčiak et al., 2017a). The KATANIN holoenzyme comprises of heterodimers of the p60 and a structural 80 kDa (p80) subunit (Hartman et al., 1998). It forms hexameric rings on the surface of microtubules and exerts its catalytic activity by ATP hydrolysis (Hartman and Vale, 1999; Stoppin-Mellet et al., 2007). The *A. thaliana* genome contains a single gene encoding for the p60 subunit and four genes encoding for different p80 subunits (Wang et al., 2017). Cellular activities of KATANIN1 include the severing of γ-tubulin-nucleated microtubules growing from the walls of pre-existing microtubules (Nakamura et al., 2010; Nakamura, 2015), severing at microtubule crossovers (Wightman and Turner, 2007; Soga et al., 2010a,b; Lindeboom et al., 2013; Zhang et al., 2013), or promoting microtubule bundle formation (Stoppin-Mellet et al., 2006).

Cellular functions of KATANIN1 in plants were studied using mutants with variable defects of the p60 subunit (Luptovčiak et al., 2017a). Phenotypic studies of *katanin1* mutants such as *botero1, ectopic root hair 3, fragile fiber2* (*fra2*)*, lue1*, and *katanin1-2 (ktn1-2*) (Bichet et al., 2001; Webb et al., 2002; Panteris et al., 2011; Panteris and Adamakis, 2012; Komis et al., 2017), showed aberrant organization of cell files in roots, exhibiting oblique cell walls. In such mutants, cytokinesis is nonetheless completed, suggesting that KATANIN1-dependent microtubule severing activity in vegetative organs might be involved in CDP orientation and in acceleration of cytokinesis (Sasaki et al., 2019). The phenotypic pleiotrophy of *katanin1* mutants is suggestive of a global importance of microtubule severing on plant development. The *fra2* mutant displays dwarf phenotype of the root (Burk et al., 2001; Luptovčiak et al., 2017a) similar to *lue1*, achieving only 30% of the wild type size (Meier et al., 2001; Bouquin et al., 2003). In addition, the *ktn1-2* mutant also exhibits defective root growth (e.g., Luptovčiak et al., 2017a), reduced fertility and abnormal formation of hypophysis during embryogenesis (Luptovčiak et al., 2017b). At the subcellular level *ktn1-2* mutant shows defective organization of mitotic microtubule arrays, delayed mitotic, and cytokinetic progression, and unstable spindle, and phragmoplast positioning (Panteris and Adamakis, 2012; Komis et al., 2017).

Although previous genetic and cell biological studies proposed a role of KATANIN1 in CDP orientation in relation to aberrant phenotypes of *katanin1* mutants, the precise cellular mechanism leading to radialy expanded root phenotypes is still unknown. Here we used advanced live cell imaging method of light-sheet fluorescence microscopy (LSFM) for non-invasive, fast, and gentle imaging of growing control and *ktn1-2* roots carrying a GFP-TUA6 microtubule marker over the course of several hours. Employing LSFM we identified ectopic longitudinal cell divisions in the calyptrogen, the procambium, and in mature parts of roots during the formation of lateral root primordia. Such ectopic divisions appear to be one of the mechanisms contributing to the radial expansion of *ktn1-2* roots, in addition to previously reported defects in cortical microtubule organization and CDP deregulation. This study shows that KATANIN1 is required for the occurrence of regular spatio-temporal cell divisions and the establishment of tissue patterning in *A. thaliana* roots.

## Materials and Methods

### Plant Material

*Arabidopsis thaliana* allelic mutants *fra2* (Burk and Ye, 2002) and *lue1* (Bouquin et al., 2003) and the knock out mutant *ktn1-2* (Nakamura et al., 2010) and wild type ecotype Columbia (Col-0) corresponding to the background of all mutants reported herein were used. Mutant *lue1* bears a nonsense mutation in the catalytic AAA domain, producing a truncated form of the protein. Mutant *fra2* bears a deletion mutation producing also a truncated version of the protein, but mutation is localized closer to the C-terminus. Produced truncated protein is thus slightly longer as compared to *lue1* mutant and possesses whole catalytic domain. *ktn1-2* is a T-DNA insertional null mutant lacking protein production (Burk et al., 2001; Meier et al., 2001; Bouquin et al., 2003; Nakamura et al., 2010; Luptovčiak et al., 2017a). For germination, Col-0 and mutant seeds were surface sterilized, plated on 0.8% w/v Phytagel® solidified ½ Murashige and Skoog medium (½ MS) with 1% w/v sucrose, stratified for 1–4 days at 4°C and subsequently transferred to environmental chamber with controlled light/dark cycle, temperature and humidity (16 h light/8 h dark, 21°C, 70% humidity). Microscopic documentation of the cell division patterns and their spatial distribution within the root, was performed with seedlings of Col-0 and *ktn1-2* mutant expressing *35S::GFP:TUA6* (Komis et al., 2017) using LSFM. The spatial organization of cortical, mitotic, and cytokinetic microtubules was documented in root meristems of the wild type line (Col-0 background) expressing *35S::mCherry:TUA5* marker (Gutierrez et al., 2009; Sampathkumar et al., 2011) using spinning disk microscopy. Visualization of microtubules in *lue1* mutant for the same analysis was performed in plants originating from crossing of Col-0 expressing *35S::mCherry:TUA5* marker with *lue1* mutant. In this case plants from F_2_ seeds were used for spinning disk microscopy analysis.

### Whole-Mount Immunofluorescence Localization of Microtubules

Seedlings of Col-0 wild type and *ktn1-2, fra2*, and *lue1* mutants were processed for whole-mount immunolocalization of microtubule arrays as described before (Šamajová et al., 2014; Smékalová et al., 2014). Briefly, after fixation and washing, seedlings were immunolabeled with rat anti-α-tubulin (clone YOL1/34; BioRad) primary antibody diluted 1:300 in 3% (w/v) BSA in PBS at 4°C overnight, followed by incubation in Alexa-Fluor 488 goat anti-rat secondary antibody diluted 1:500 in PBS containing 3% (w/v) BSA at 37°C for 1.5 h and at room temperature for 1.5 h. After mounting in antifade mounting medium samples were imaged with a spinning disk microscope (Cell Observer SD, Carl Zeiss, Germany) equipped with EC Plan-Neofluar 40×/1.3 NA (Carl Zeiss, Germany) objective, excitation laser line 488 nm, and emission filter BP525/50 for fluorescence detection.

### Microscopy

#### Wide-Field Microscopy

To address root structure and particularly the arrangement of the cell stem niche, primary root tissue organization, formation of lateral root primordia, and emergence of lateral roots, seedlings were fixed in 50% v/v ethanol and 10% v/v acetic acid and subsequently cleared in chloral hydrate solution (chloral hydrate:glycerol:water, 8:3:1). Starch grains in root columella cells were stained with Lugol solution (Sigma) prior to clearing to better discriminate development and organization of columella cell layers. These samples were examined with DIC-equipped Plan-Neofluar 10×/0.3 NA and Plan-Neofluar 40×/0.75 NA objectives of a Zeiss AxioImager M2 microscope and documented with a Zeiss AxioCam ICm1 camera.

#### Confocal Laser Scanning Microscopy

For imaging of cell file organization in root epidermis, living roots were stained with 4 μM of the styryl dye FM4-64 in liquid ½ MS medium in order to delineate cell borders and documented with a Zeiss LSM710 microscope. Plants were observed with Plan-Apochromat 20×/0.8 NA objective and data sets were analyzed with Zeiss Zen 2014 software (Blue Version).

#### Spinning Disk Microscopy

Cortical and mitotic microtubules in root apical meristem of wild type Col-0 and *lue1* mutant expressing *35S::mCherry:TUA5* marker were imaged using Cell Observer Z1 spinning disk confocal microscope (Carl Zeiss, Germany). Seedlings were placed to drop of ½ MS medium on microscopy slide delineated by spacers on the side to form imaging microchamber after covering with a coverslip. Roots of the seedlings were sandwiched between slide and coverslip, while green parts of the seedlings were outside of the chamber and in contact with air during imaging. Seedlings were examined with an EC Plan-Neofluar 40×/1.3 NA oil immersion objective. Fluorescence signal was excited with laser line 488 nm and detected using emission filter BP525/50 in high-resolution Evolve 512 back-thinned EM-CCD camera (Photometrics). Acquired data were analyzed with Zeiss Zen 2014 software (Blue Version).

#### LSFM

Seedlings expressing *35S::GFP:TUA6* germinating in Phytagel® solidified ½ MS medium were installed into fluorinated ethylene propylene (FEP) tubes with an inner diameter of 2.8 mm and wall thickness of 0.2 mm (Wolf-Technik, Germany) according to published protocol (Ovečka et al., 2015). Briefly, seedlings were placed inside of the FEP tube in a way supporting root growth in the block of the culture medium in the bottom part of the FEP tube, while upper part of the FEP tube containing air accommodated green part of the seedling. Plantlets developing in the FEP tube during imaging had thus access to air. Samples were placed into the observation chamber of the light-sheet microscope tempered to 22°C using a Peltier heating/cooling system. Observation chamber was filled with a liquid ½ MS medium, which was sterilized using a sterile syringe filter. Long-term live-cell imaging of plants was done with the light-sheet Z.1 fluorescence microscope (Carl Zeiss, Germany) equipped with W Plan-Apochromat 20×/1.0 NA imaging objective (Carl Zeiss, Germany) and two LSFM 10×/0.2 NA illumination objectives (Carl Zeiss, Germany). Samples were imaged using dual-side light-sheet illumination with excitation laser line 488 nm, beam splitter LP 560 and with emission filter BP505-545. For recording of images the PCO.Edge sCMOS camera (PCO AG, Germany) was used and the exposure time was set up to 30 ms with imaging every 5 min. Observation periods spanned between 6–10 h.

#### Image and Statistical Analysis

Images acquired in individual z-stacks from light-sheet microscope were joined together using maximum intensity projection function of the Zeiss Zen 2014 software (Black Version). Maximum intensity projection of all acquired z-stacks projected along to time dimension were used for generation of movies, reflecting dynamics of the root growth and cell divisions during the indicated time periods. Quantitative parameters defining ectopic cell positions, ratio of longitudinal, and transverse cell divisions, the number of protoxylem files, number and cellular density of lateral root primordia were calculated directly from the microscopy images. Stages of lateral root primordia (LRP) development were annotated according to Malamy and Benfey (1997), based on the cell division pattern as well as histological and anatomical characteristics. In particular, we characterized the stage I with anticlinal cell divisions in the pericycle forming one-layered LRP, the stage II with anticlinal, and periclinal cell divisions forming the second layer of the LRP, the stage III where periclinally dividing cells generated a three-layered LRP, the stage VI in which the LRP possessed distinct layers of the cortex and the epidermis surrounding an internal stelar tissue, and the stage of LRP emergence from the main root. Kymographs were generated using Zeiss Zen 2014 software (Blue Version). All parameters were compared by 2-tailed Student's *t*-test with statistical significance assessed at least at the level of *p* < 0.05.

## Results

### Disturbed Primary Root Phenotypes of *katanin1* Mutants

**Figure 1:**
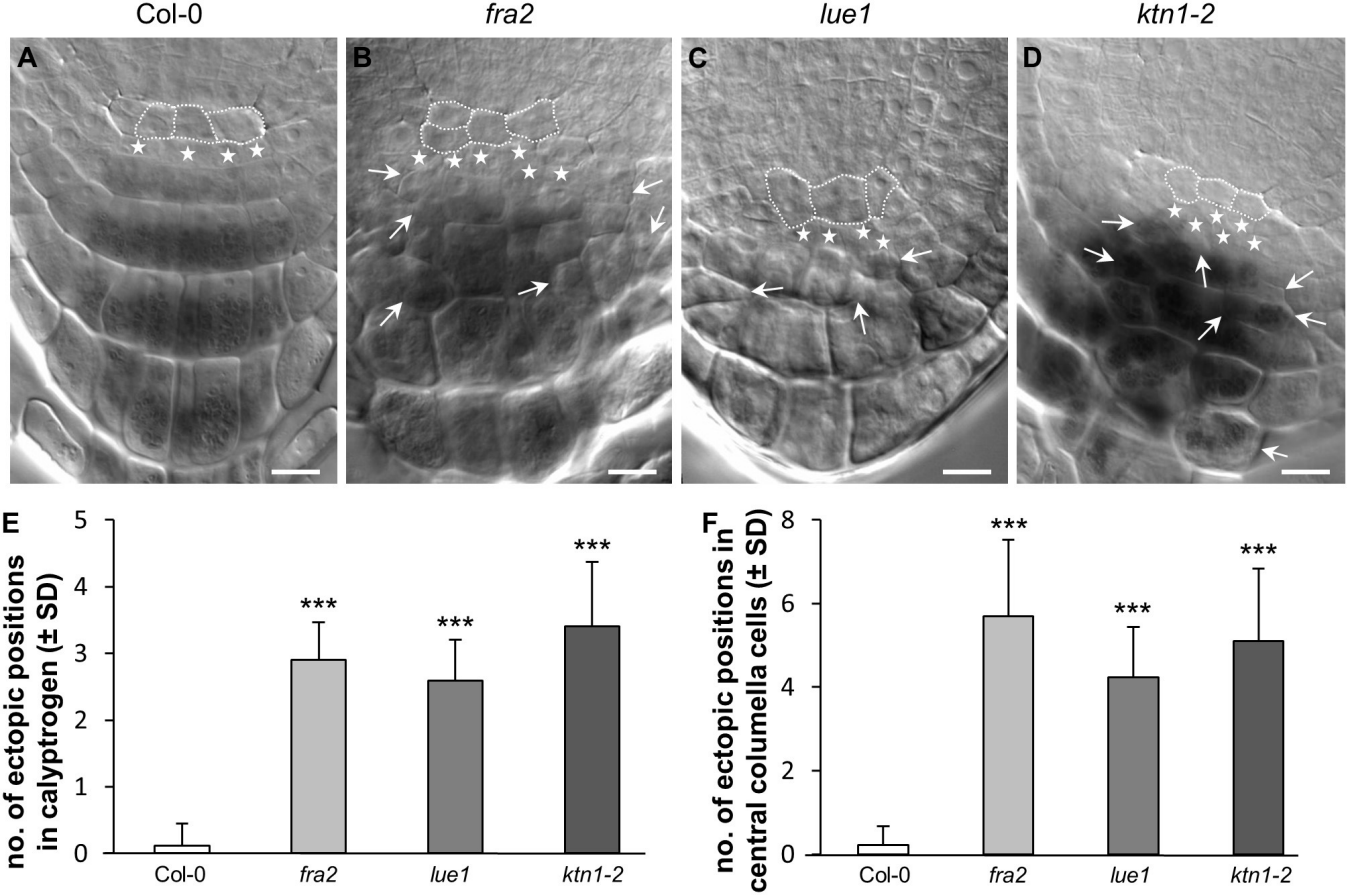
Ectopic cell divisions in root calyptrogen and root cap malformations. **(A–D)** Organization of quiescent center and root cap in the root apex of Col-0 **(A)**, *fra2* **(B)**, *lue1* **(C)**, and *ktn1-2* **(D)** mutants. Cells of the quiescent center are outlined by white dashed line, cells of the columella initials (calyptrogen) are indicated by white stars. Ectopic positions of differentiated cells in the columella are indicated by arrows. **(E,F)** Quantitative evaluation of ectopicaly positioned cells in the tiers of columella initials—calyptrogen **(E)** and in columella central cells **(F)**. Data for quantification were collected from 9–17 seedlings. Asterisks indicate statistical significance between measurements (***p < 0.001). Bar: **(A–D)** 10 μm.

The root architecture at the primary root tip region was examined in detail in order to gain insight into the cell patterning and tissue organization. Quiescent center cells (QC), columella meristematic cells (calyptrogen), and root cap cells in the primary root apex of Col-0 wild type, *fra2, lue1*, and *ktn1-2* mutants were examined in fixed and optically-cleared preparations (Figure 1 and Figure S1). In Col-0 both QC and calyptrogen cells were orderly arranged (Figure 1A) showing regular patterning of the calyptrogen (Figures 1A,E) and central columella cell (Figures 1A,F) organization. Calyptrogen in Col-0 consisted of four small meristematic cells, typically arranged in one row within the median optical section of the root (Figure 1A). This pattern was highly stable in Col-0 (Figures 1E). Regular arrangement of central columella cells in four distinct cell layers in Col-0 (Figures 1A,F, Video S1) was supported and determined by their uniform rectangular shape, resulting from synchronized periclinal cell divisions of the founding calyptrogen cells. An overview of *fra2, lue1*, and *ktn1-2* showed that these regular arrangements are severely disturbed, resulting in aberrant organization of cell divisions in the QC and the calyptrogen of all three mutants (Figures 1B–D). Quantitatively, aberrant cell divisions and CDP orientations, which were measured in calyptrogen (Figure 1E; *n* = 9–17), correlated with the observed ectopically positioned cells in the central columella (Figure 1F; *n* = 9–17). However, *fra2, lue1*, and *ktn1-2* mutants possess also higher number of calyptrogen cells indicating that the ectopic cell divisions occurred in this tissue (Figures 1B–D). Quantitative evaluation revealed that all the three *katanin1* mutants show significantly higher numbers of ectopic cell positions, which means extra cells at specific positions, like calyptrogen (Figure 1E), and central columela cells (Figure 1F), which disturb organized layered patterning. Importantly, the highest number of ectopic cell positions in calyptrogen was observed in the *ktn1-2* mutant (Figure 1E, Video S2). Furthermore, changes of ordered cell division pattern in calyptrogen of *katanin1* mutants affected considerably the general organization of starch-containing central columella region, showing failure in the typical layered structure (Figure S1).

**Figure 2:**
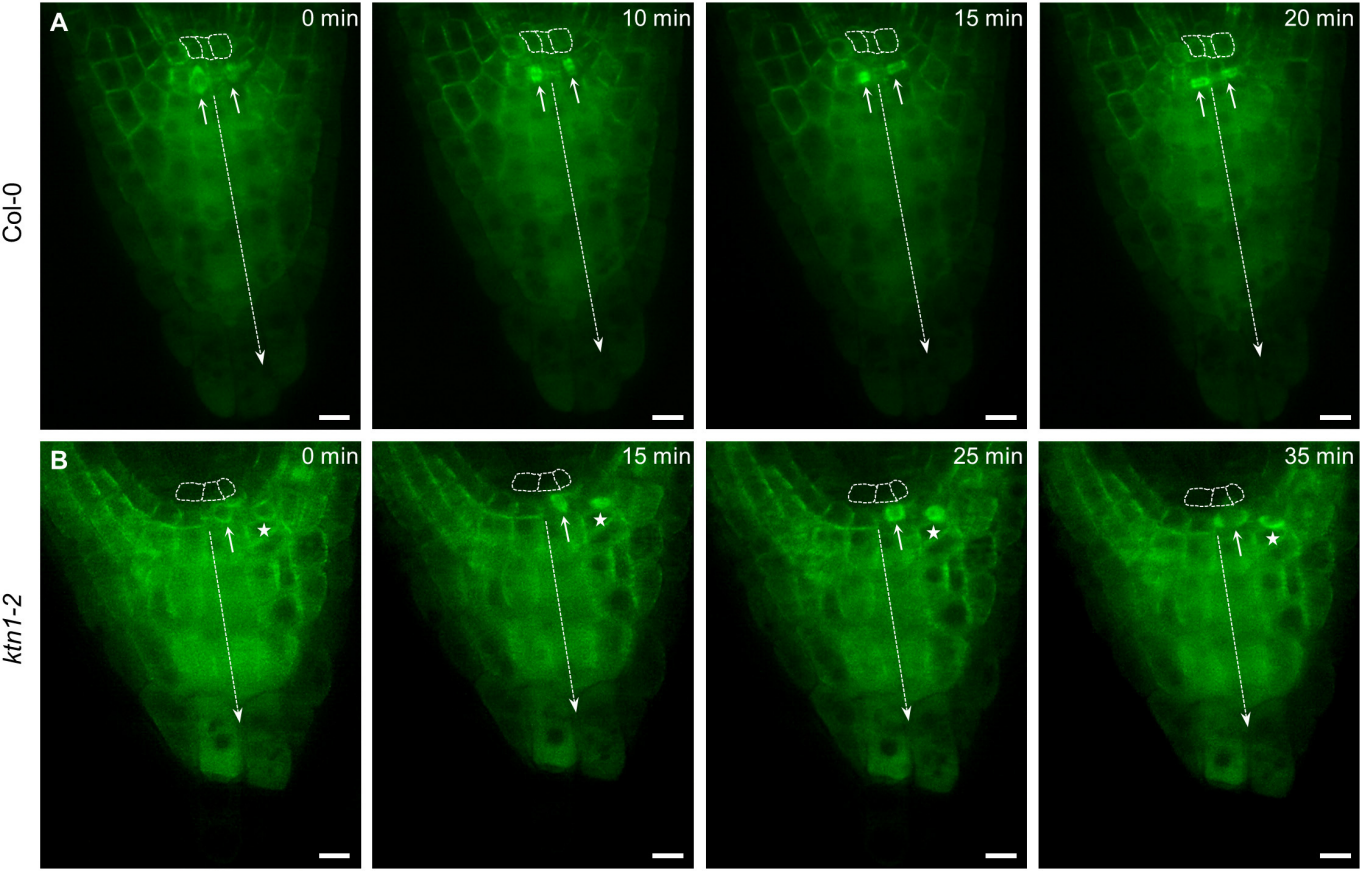
Cell divisions in root columella stem cells (calyptrogen) of Col-0 and *ktn1-2* mutant plants stably expressing a GFP-TUA6 microtubule marker. **(A)** Synchronized proliferative cell divisions (arrows) of columella stem cells in the root apex of Col-0 (*n* = 3). **(B)** Asynchronous proliferative cell division (arrow) on one side of the calyptrogen in the root apex of *ktn1-2* mutant (*n* = 3). Simultaneous joined cell division of lateral root cap cell (star) beneath of epidermis/lateral root cap initial. Cells of the quiescent center are outlined by dashed line, axis of the columella is indicated by dashed arrow. Time progression of the cell division is indicated in min. Bars: **(A,B)** 10 μm.

Previous studies using tubulin immunolocalization showed changes in organization of cortical microtubules in root cells of *katanin1* mutants (Takáč et al., 2017; Vavrdová et al., 2019). By using a whole mount immunofluorescence localization method we confirmed predominantly transverse and parallel organization of cortical microtubules in root epidermal cells of Col-0 wild type (Figures S2A,E), while less ordered transverse or randomly oriented cortical microtubules were observed in root epidermal cells of *fra2* (Figure S2B) *lue1* (Figure S2C) and *ktn1*-*2* (Figures S2D,F) mutants. To study cell division patterns including cell division synchronization in growing wild-type and *katanin1* mutants we used Col-0 and *ktn1-2* mutant plants stably expressing GFP-TUA6 marker applying minimally invasive light-sheet fluorescence microscopy (LSFM). LSFM is mesoscopic imaging method providing lower axial resolution as compared to confocal laser scanning microscopy. Therefore, it is not regularly used for acquisition of crisp images of microtubules. However, in epidermal and lateral root cap cells of both Col-0 and *ktn1-2* mutant plants stably carrying GFP-TUA6 marker, cortical, and mitotic microtubules can be visualized (Figure S3, Videos S3–S6). Importantly, LSFM is suitable for deep tissue imaging in the whole plant organs, allowing to visualize cell size, cell shape, and spatial distribution of dividing cells in the whole root apex of both Col-0 (Video S1) and *ktn1-2* mutant (Video S2) plants carrying GFP-TUA6 marker. The microscopic analysis of root growth recorded over the period of 3 h revealed synchronization of the proliferative cell divisions in the calyptrogen of the root apex in Col-0 (Figure 2A, arrows, Video S7). On the contrary, asynchronous proliferative cell divisions were detected in the root apex of *ktn1-2* mutant (Figure 2B, arrow, Video S8). In addition, we observed cell divisions of lateral root cap cells beneath the epidermis/lateral root cap initial that occured synchronously and in close proximity with cell divisions in the calyptrogen of *ktn1-2* mutant (Figure 2B, star).

Cell patterning of epidermis in the primary root was also examined. By delineating root cells of Col-0 wild-type and *fra2, lue1*, and *ktn1-2* mutants with FM4-64, respectively, it was possible to observe aberrant positioning of epidermal cells in all three mutants. Col-0 epidermal cells were orderly arranged (Figure S4A) showing only occasionally oblique CDPs (ca. 4%; Figure S4E). In contrast, epidermal cell patterning was disturbed in *fra2, lue1*, and *ktn1-2* mutants by frequent oblique CDPs (Figures S4B–E), which were significantly increased compared to Col-0 (around 20% of oblique CDPs in all mutants examined).

Organization of microtubules in the root apex cells of *lue1* mutant was compared to Col-0 using a mCherry-TUA5 microtubule marker (Figure S5). Microtubules in both dividing and non-dividing root meristematic cells of Col-0 were normaly organized, with perpendicular orientation of PPBs and phragmoplasts, as well as parallel orientation of mitotic spindles in respect to the root longitudinal axis (Figures S5A–E). The structure of the PPB in *lue1* mutant was sometimes incomplete, loosely organized, and occasionally consisting of several individual rings (Figures S5F,G, arrows). We observed oblique and shifted CDPs in the epidermis and central columella cells of the root cap (Figures S5H–J, arrows), but also the presence of tilted and abnormally positioned PPBs in some meristematic cells of *lue1* mutant (Figures S5K–M, arrows).

**Figure 3:**
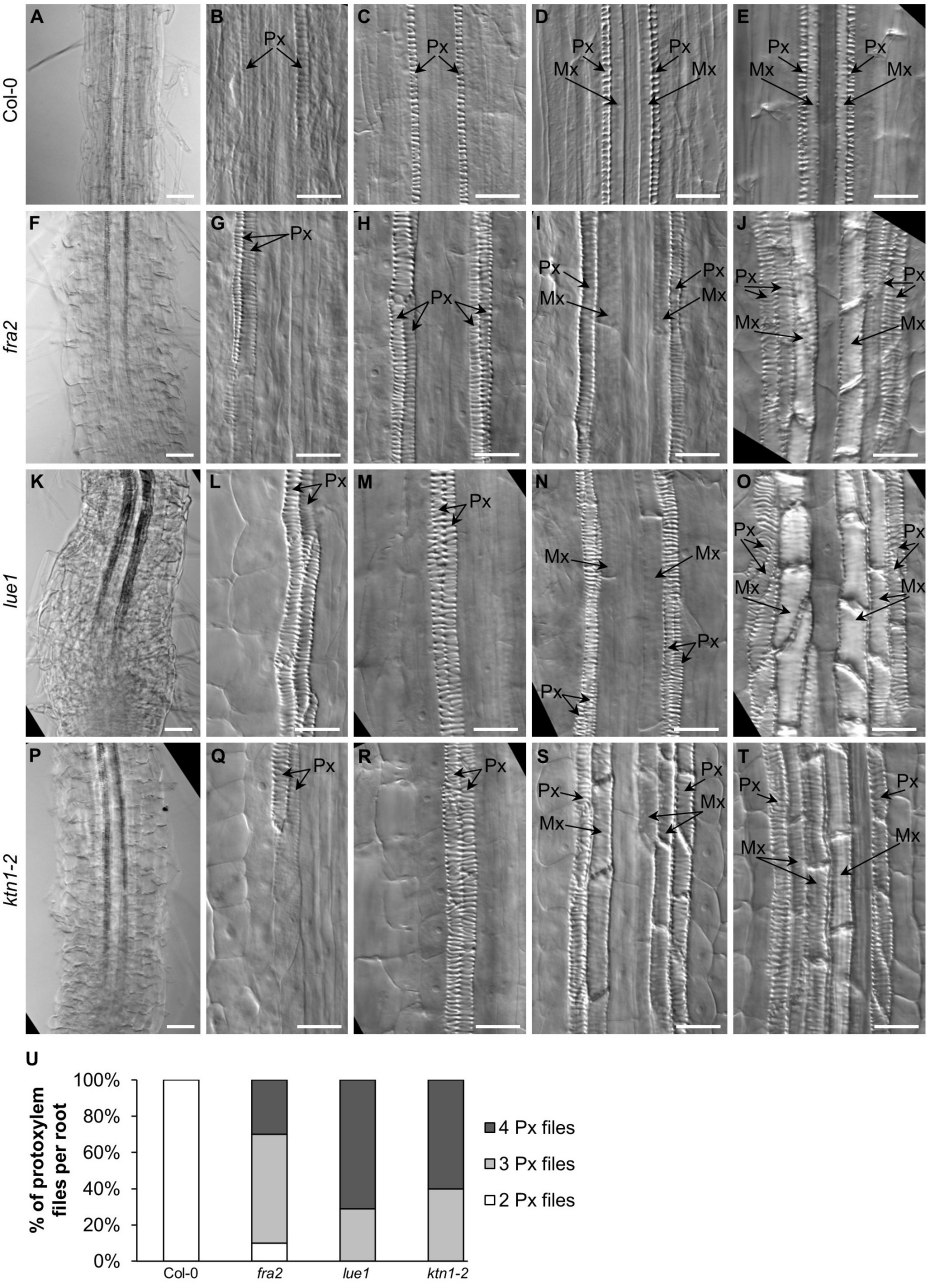
Development and organization of xylem vascular tissue in primary roots of Col-0 and *katanin1* mutants. **(A–U)** Differentiation and spatial organization of root protoxylem (Px) and metaxylem (Mx) vascular elements at different distances from the root tip in Col-0 **(A–E)**, *fra2* **(F–J)**, *lue1* **(K–O)**, and *ktn1-2* **(P–T)** plants. Position of protoxylem (Px) and metaxylem (Mx) elements is indicated by arrows. **(U)** Frequencies of 2, 3, or 4 differentiated protoxylem cell files in mature part of examined roots in Col-0 and *katanin1* mutant seedlings (*n* = 9–11). Plants for analysis were 7 days old. Bars: **(A,F,K,P)** 50 μm; **(B–E,G–J,L–O,Q–T)** 20 μm.

Primary xylem formation was also affected in all three mutants (Figure 3). Typically, protoxylem and metaxylem elements of Col-0 primary root consist of two single cell files each (and three files of metaxylem elements at later stages) organized into juxtaposed pairs with the protoxylem elements pointing outwards and the metaxylem elements pointing inwards the central cylinder (Figures 3A–E). In *fra2* (Figures 3F–J), *lue1* (Figures 3K–O), and *ktn1-2* (Figures 3P–T), protoxylem organization was affected, as it was found to comprise from more than two cell files (Figure 3U).

The process of protoxylem cell file duplication can be developmentally tracked in the central procambium meristem zone (Figure S6). In the primary root apex of *ktn1-2* mutant we observed cell file(s) originating from procambium initials consisting of cells with abnormal size, shape, orientation, and position. The cell division pattern was disturbed by ectopic *T*-shaped longitudinal cell divisions (Figure S6A, arrow), which led to reorganization of the cell file and the duplication of protoxylem files (Figures S6A,B, arrowheads).

**Figure 4:**
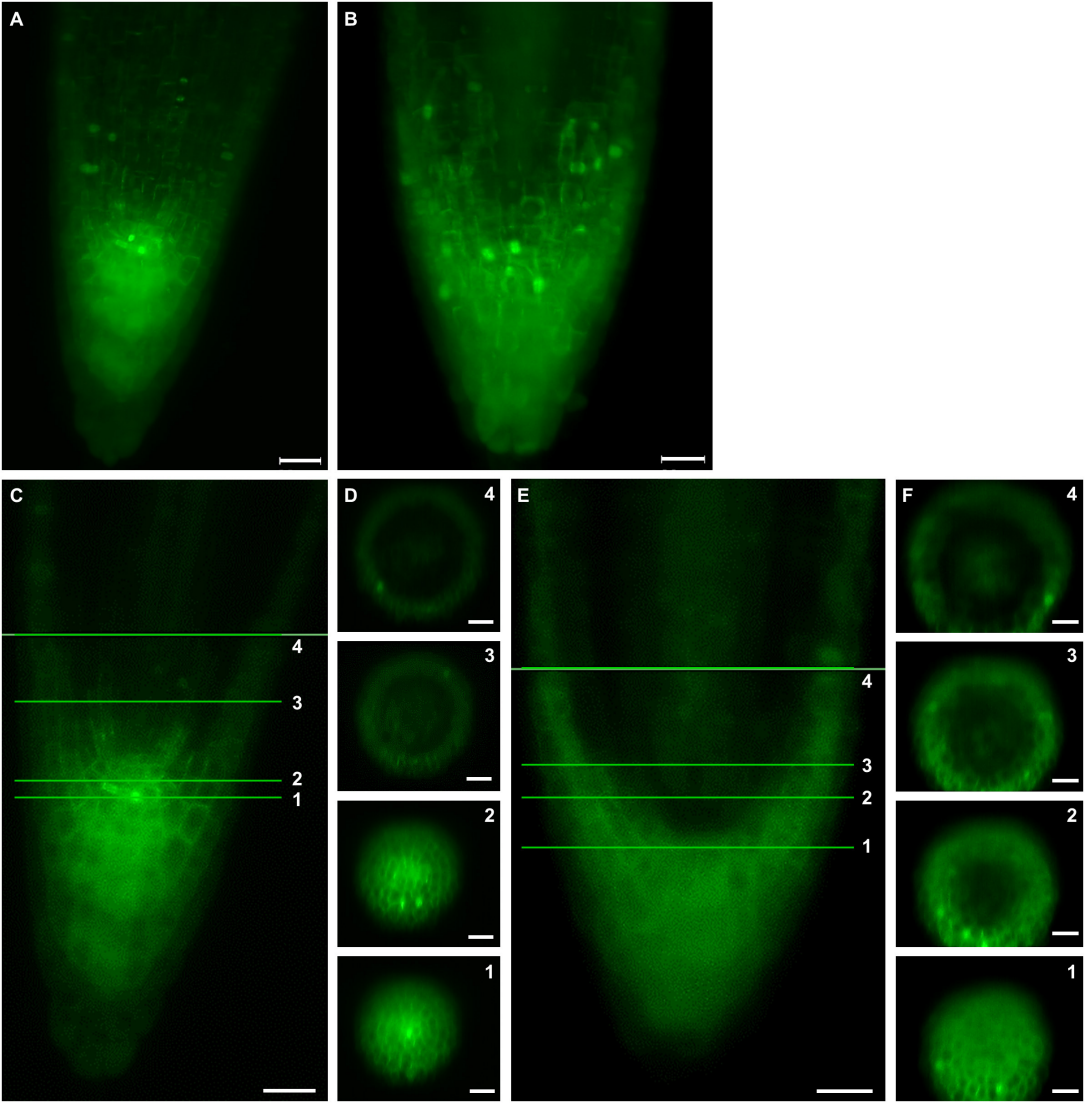
Spatial distribution of cell divisions in meristem and root cap of primary root in Col-0 **(A,C,D)** and *ktn1-2* mutant **(B,E,F)**. Analyzed plants stably expressed a GFP-TUA6 microtubule marker. **(A,B)** Volume rendering of a whole root tip showing total number of cell divisons at certain time point in primary root tip of Col-0 **(A)** and *ktn1-2* mutant **(B)**. **(C,E)** Median sections of primary root tip of Col-0 **(C)** and *ktn1-2* mutant **(E)**. **(D,F)** Orthogonal projections in calyptrogen (1), quiescent center (2), and two other zones (3, 4) in primary root meristem. Bars: **(A,B)** 20 μm; **(C,E)** 50 μm; **(D,F)** 100 μm.

Live cell imaging of growing root tips using LSFM offers the opportunity to characterize the spatial distribution of cell divisions both in the root meristem and in the root cap. Frequency, orientation and distribution of cell divisions can be tracked using plant lines carrying GFP-TUA6 microtubule marker. Thus, division activity in the root cap and the meristematic part of the root were monitored in Col-0 (Figure 4A, Video S7). These division activities were broadly redistributed and ectopically positioned in the *ktn1-2* mutant (Figure 4B, Video S8). Orthogonal projections through the whole root apex at the position of the stem cell niche and at additional three positions within the root meristem revealed few cell divisions at the peripheral meristem and in the root cap of the Col-0 (Figures 4C,D, Video S7), while more frequent cell divisions were observed in the same positions of the *ktn1-2* mutant (Figures 4E,F, Video S8). The higher frequency of ectopic cell divisions located in the peripheral meristem and the root cap of the *ktn1-2* mutant was particularly evident after data processing using 3-D rendering of the whole root apex (Figures 4A,B).

**Figure 5:**
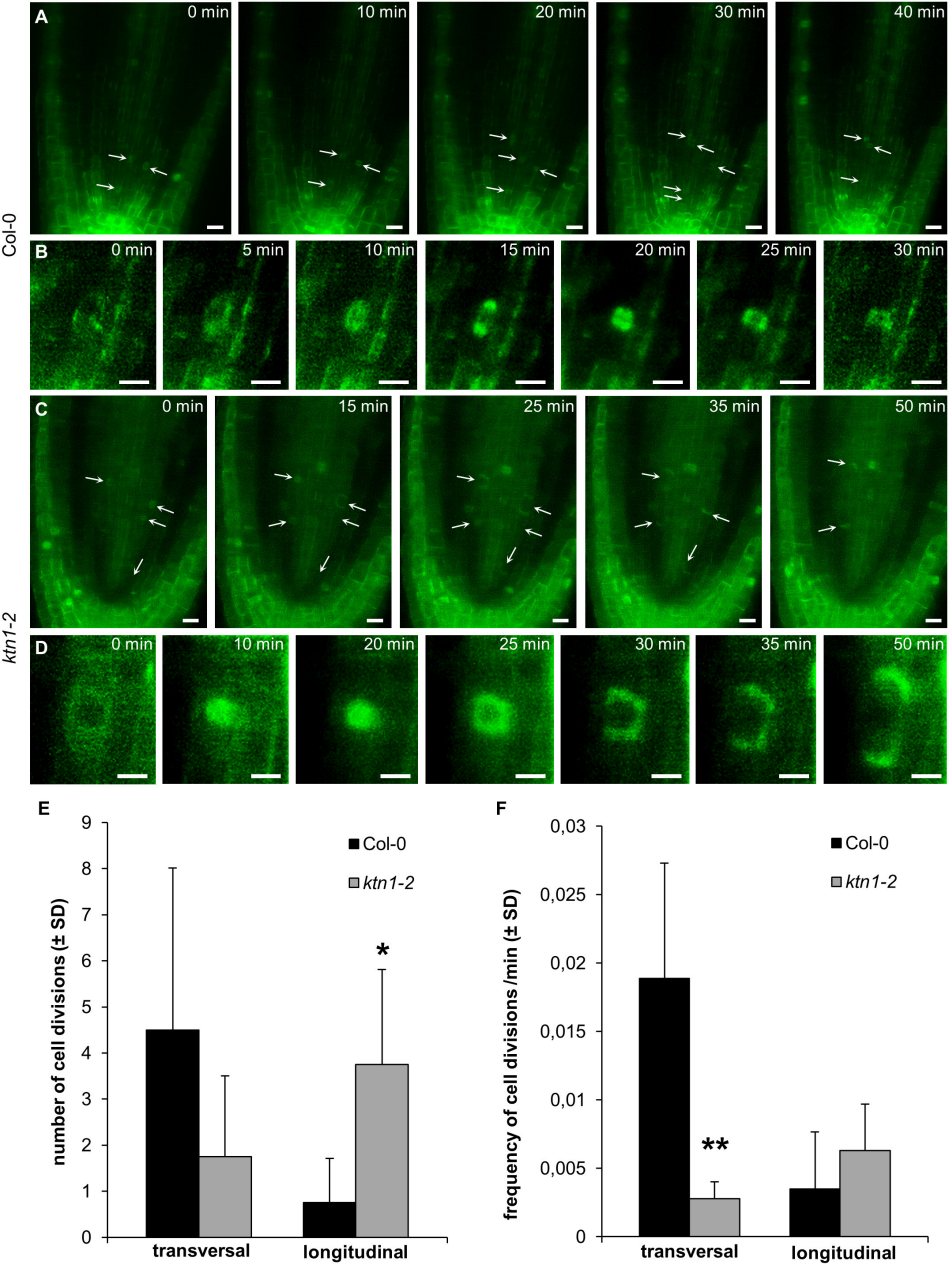
Cell divisions in procambium of the primary root apex of plants stably expressing a GFP-TUA6 microtubule marker. **(A,B)** Proliferative transversal cell divisions in procambium of Col-0 (arrows). Progression of one representative cell division is depicted in **(B)**. **(C,D)** Longitudinal cell divisions in procambium of *ktn1-2* mutant (arrows). Progression of one representative cell division is depicted in **(D)**. **(E,F)** Absolute numbers **(E)** and frequency/min **(F)** of proliferative transversal and longitudinal cell divisions in procambium of Col-0 and *ktn1-2* mutant. Calculations are based on data collection from 4 seedlings. Asterisks indicate statistical significance between measurements (*p < 0.05, **p < 0.01). Bars: **(A,C)** 10 μm; **(B,D)** 5 μm.

To reveal the typical pattern of cell divisions in central root part of the *ktn1-2* mutant, we analyzed the frequency of proliferative (transversal) and longitudinal cell divisions in procambium of the primary root apex using stably expressed GFP-TUA6 microtubule marker. Proliferative transversal cell divisions in procambium of the Col-0 root meristem characterized by phragmoplast mid-zone orientated perpendicularly to the root axis are depicted in Figures 5A,B. Notably, we frequently observed longitudinal cell divisions with phragmoplast mid-zone oriented in parallel to the root axis (both radially or tangentially oriented in respect to the root surface) in the procambium of the *ktn1-2* mutant (Figures 5C,D). Quantitative comparison revealed statistically significant reduction in the frequency of proliferative transversal cell divisions and the higher number of longitudinal cell divisions in the *ktn1-2* mutant (Figures 5E,F). These data suggest that the higher ratio of ectopic longitudinal cell divisions in the procambium of *ktn1-2* root meristem leads to the increased number of cell files in this tissue.

### Abnormal Patterning During Lateral Root Formation

**Figure 6:**
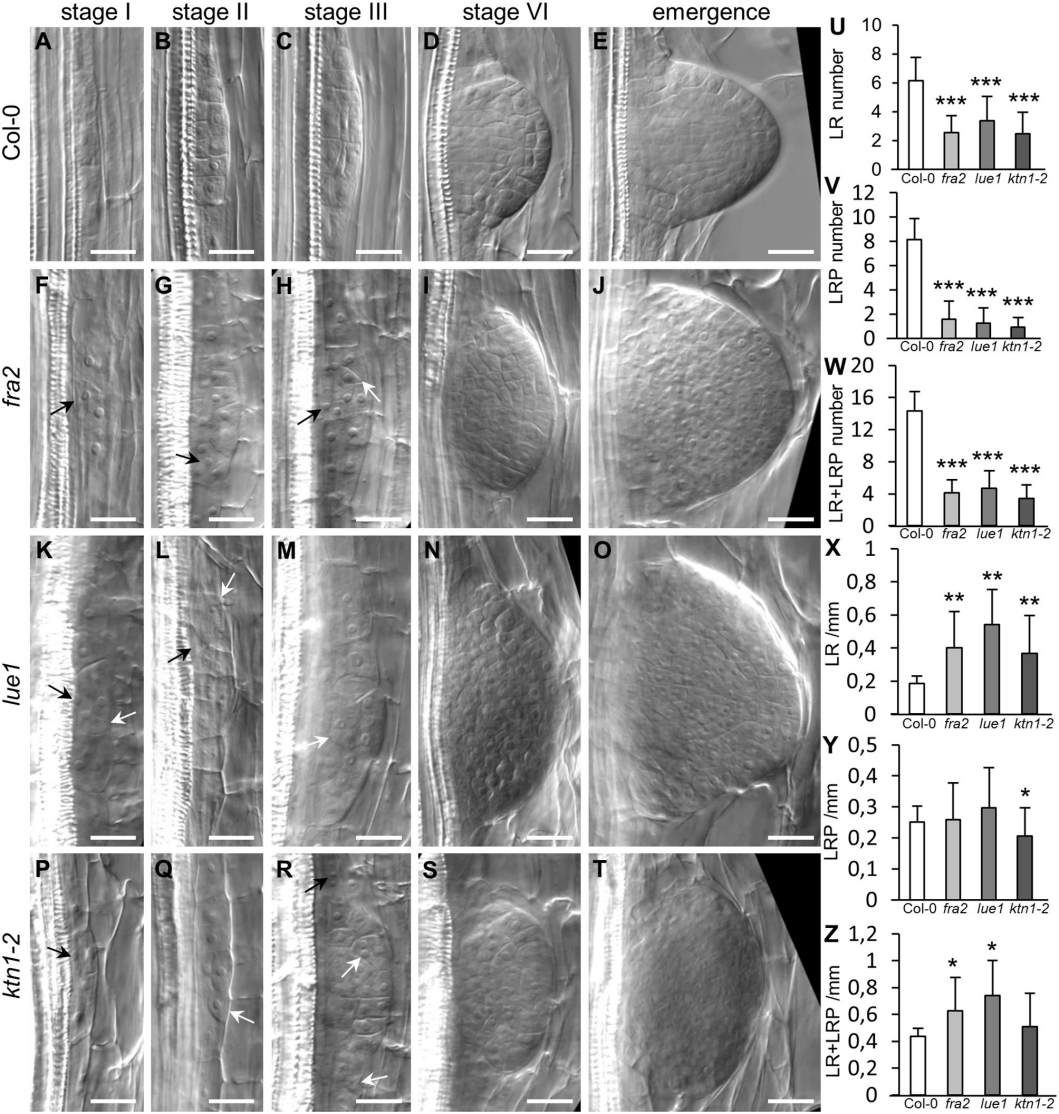
Phenotypes of lateral root primordia in Col-0 and *katanin1* mutants. **(A–T)** Representative pictures of selected developmental stages of lateral root primordia in 7 days old seedlings of Col-0 **(A–E)**, *fra2* **(F–J)**, *lue1* **(K–O)**, and *ktn1-2* **(P–T)** mutants. Ectopic positions of cells in early stages of lateral root primordia development are indicated by arrows. Stages of lateral root primordia development were annotated according to Malamy and Benfey (1997). **(U–Z)** Quantitative analysis (±SD) of lateral root phenotype in 7 days old seedlings of Col-0 and *katanin1* mutants. Number of lateral roots (LR) per plant **(U)**, number of lateral root primordia (LRP) per plant **(V)**, number of lateral roots, and lateral root primordia (LR + LRP) per plant **(W)**, density of lateral roots (LR/mm; **X**), density of lateral root primordia (LRP/mm; **Y**), density of lateral roots, and lateral root primordia (LR+LRP/mm; **Z**). Calculations are based on data collection from 13–19 seedlings. Asterisks indicate statistical significance between measurements (*p < 0.05, **p < 0.01, and ***p < 0.001). Bar: **(A–T)** 20 μm.

Together with primary root patterning and growth, lateral root (LR) development was also severely compromised in the *katanin1* mutants. By studying cleared wild type (Col-0) roots, we were able to discern typical stages of lateral root primordium (LRP) formation and development as previously published (Malamy and Benfey, 1997). Whereas, it was possible to identify asymmetric anticlinal divisions in the pericycle during the establishment of LRP in Col-0 roots (Stage I; Figure 6A), followed by successive anticlinal and periclinal divisions leading to the LRP outgrowth in a series of stages (Figures 6B–E), in all *katanin1* mutants LRP formation was disturbed at the earliest stages, as we observed unusual oblique cell divisions in the pericycle. This resulted in ectopic positioning of several cells during LRP patterning leading ultimately to malformed emerging lateral roots (Figures 6F–J for *fra2*; Figures 6K–O for *lue1* and Figures 6P–T for *ktn1-2*). In quantitative terms we analyzed the absolute numbers of LRPs and LRs, and found out significantly reduced numbers of both in all three *katanin1* mutants (Figures 6U–W). On the other hand, linear densities of both LRPs and LRs were significantly increased as compared to Col-0 (Figures 6X–Z).

**Figure 7:**
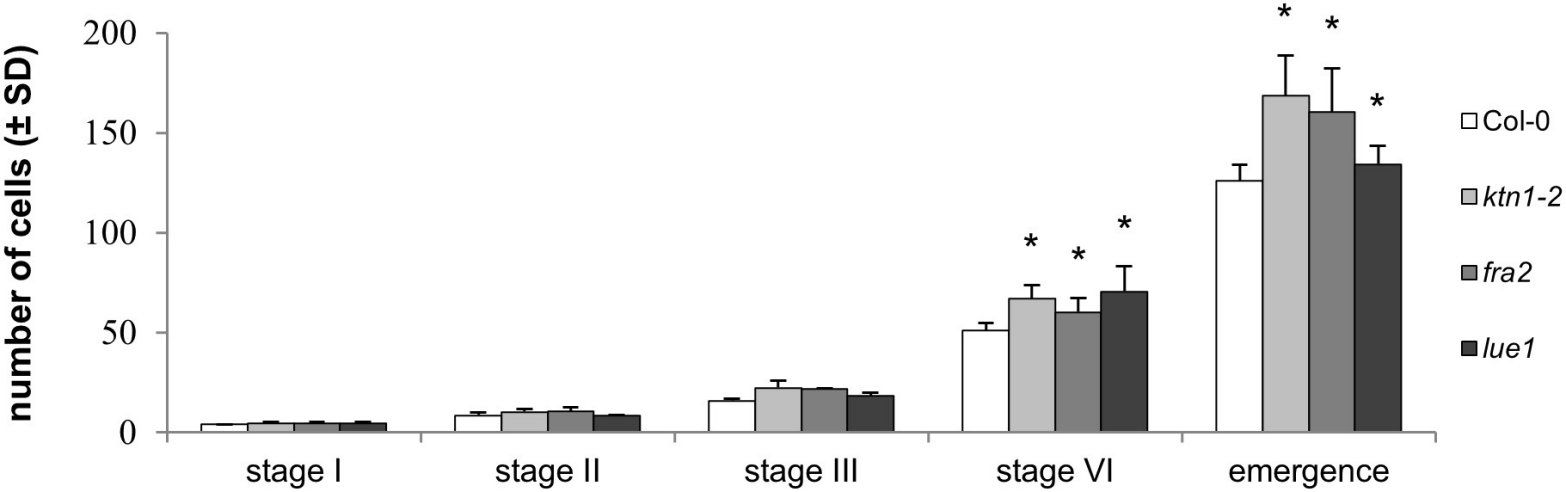
Number of cells in different stages of lateral root primordia development in Col-0 and *katanin1* mutants. Number of cell in individual developmental stages of lateral root primordia in 7 days old seedlings showing comparison between Col-0, *fra2, lue1*, and *ktn1-2* mutants. Calculations are based on data collection from 8–9 seedlings. Asterisks indicate statistical significance between measurements (*p < 0.05).

The number of cells participating at different stages of lateral root primordia development in Col-0 and *katanin1* mutants gradually increased (Figure 7). Although there were no differences in the number of cells counted from median optical sections at early stages of LRP formation (namely in stages I and II) between Col-0 and *ktn1-2*, the number of cells was significantly increased at the later stage VI of LR developement and during LR emegence in all three *katanin1* mutants (Figure 7). Moreover, detailed comparative quantitative analysis of Col-0 and *katanin1* mutants revealed that during LR formation, particularly at later developmental stages, there is an acceleration of cell divisions leading to excessive numbers of cells in the LR of *katanin1* mutants (Figure 7).

**Figure 8:**
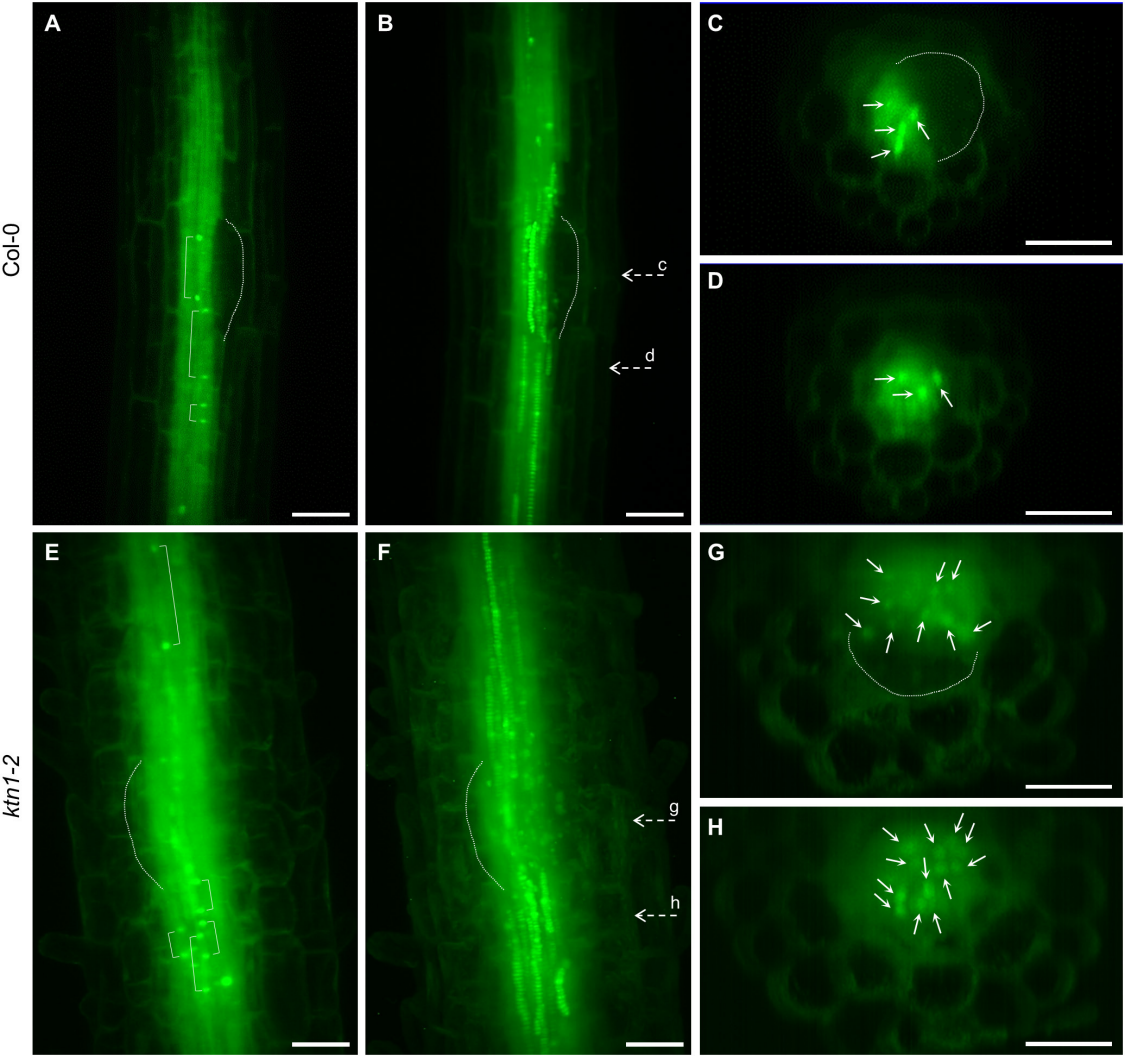
Longitudinal cell divisions in the central cylinder at the region of first lateral root primordium formation in Col-0 and *ktn1-2* mutant plants stably expressing a GFP-TUA6 microtubule marker. **(A–H)** Longitudinal cell divisions in Col-0 **(A–D)**, and *ktn1-2* mutant plants **(E–H)** in the median section **(A,E)**, maximum intensity projection **(B,F)**, and orthogonal projections **(C,D,G,H)** corresponding to dashed arrows marked with small letters (c, d, g h) in **(B,F)**. Brackets show positions of the margins of late phragmoplasts **(A,E)**. Primordia are outlined by dashed line **(A–C,E–G)**. Bars: **(A–H)** 50 μm.

**Figure 9:**
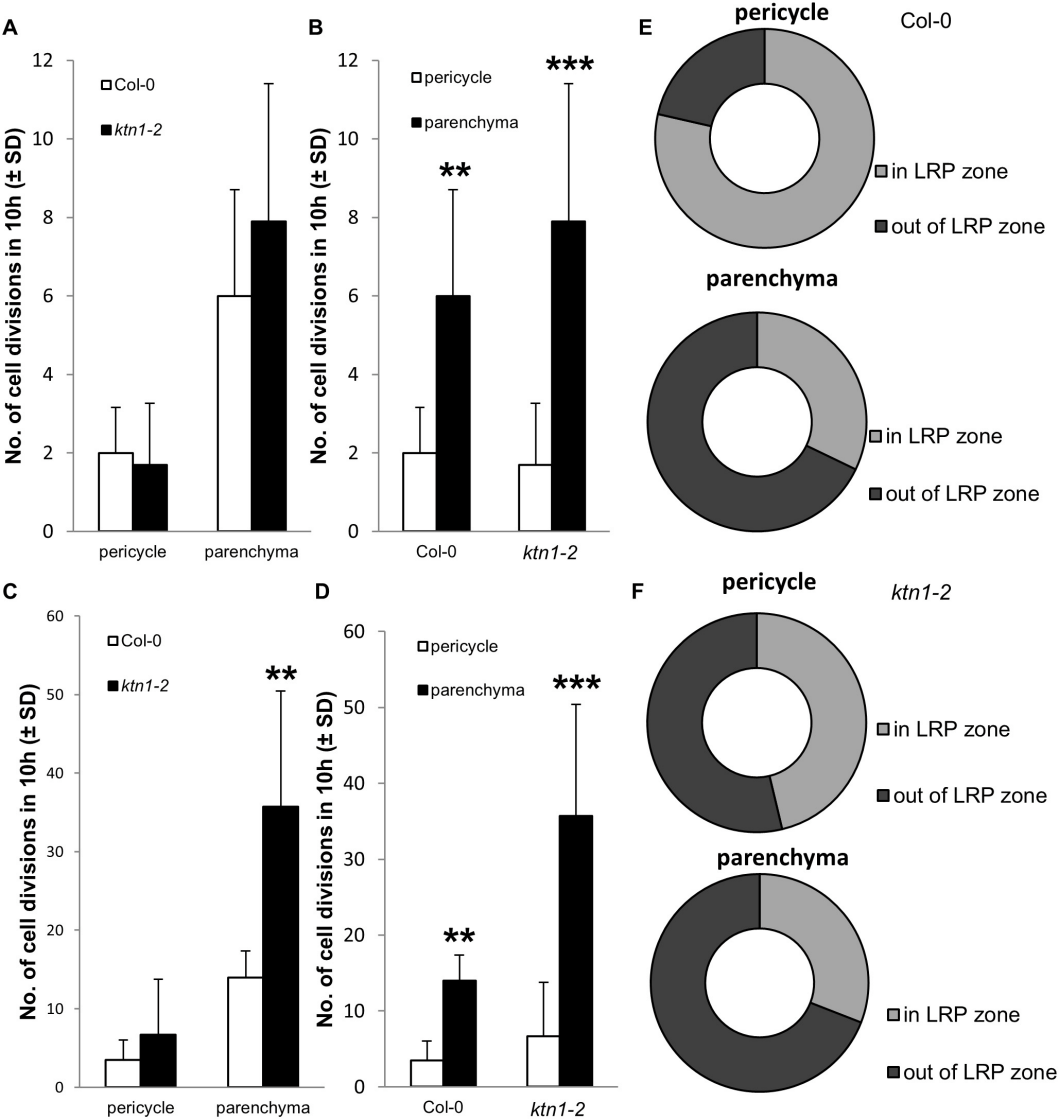
Proliferative transversal and longitudinal cell divisions in central cylinder of primary roots in Col-0 and *ktn1-2* mutant. **(A,B)** Number of proliferative transversal cell divisions in pericycle and stelar parenchyma, which is increasing the number of cells in individual cell files, in Col-0 and *ktn1-2* mutant. **(C,D)** Number of longitudinal cell divisions in pericycle and stelar parenchyma, which is increasing the number of cells or cell files in radial pattern of the central cylinder, in Col-0 and *ktn1-2* mutant. **(E,F)** Ratio of longitudinal cell divisions occuring in the central cylinder at the place of LRP formation (LRP zone) and outside of the place of the LRP formation (out of LRP zone), quantified separately for pericycle and stelar parenchyma in Col-0 **(E)** and *ktn1-2* mutant **(F)**. Cell divisions were counted in pericycle and stelar parenchyma cells in mature part of the primary root at the place of first lateral root primordium establishment within the field of view of 438.5 × 438.5 μm and time period of 10 h in roots of 5 days old seedlings. Calculations are based on data collection from 102 dividing cells of 4 Col-0 seedlings and 520 dividing cells of 7 *ktn1-2* seedlings. Asterisks indicate statistical significance between measurements (**p < 0.01, and ***p < 0.001).

Next, we addressed the question of what is the origin of the observed increased numbers of cells participating in the LRP by examining the cell division frequency in root central cylinder. In this context, we analyzed the frequencies of longitudinal cell divisions in the central cylinder of the root region at the first LRP. By using LSFM we were able to record scarce longitudinal cell divisions in parenchymatic cells of the central cylinder of Col-0, occuring both inside and outside of the LRP position (Figures 8A–D, Videos S9, S10). Similar cell divisions occured also in the parenchymatic cells of the central cylinder of the *ktn1-2* mutant, but in higher frequencies (Figures 8E–H, Videos S11, S12). Quantitative analysis revealed the ratio between proliferative-transversal and longitudinal cell divisions in the central cylinder. It was apparent that the frequencies of both proliferative transversal and longitudinal cell divisions were higher in stelar parenchymatic cells than in pericycle cells (Figures 9A–D). Importantly, the frequency of longitudinal cell divisions was significantly higher in the *ktn1-2* mutant (Figures 9C,D). In the pericycle of Col-0 the majority of longitudinal cell divisions was observed in the LRP zone (Figure 9E), however, in the *ktn1-2* mutant higher frequencies were observed both inside and outside ofthe LRP zone (Figure 9F). These data suggest that the higher frequency of such specialized cell divisions increased the number of cell files resulting in the increased radial expansion of the root in the *ktn1-2* mutant. Whereas, in Col-0 the longitudinal cell divisions were specifically located to LRP zone, in the *ktn1-2* mutant were rather uniformly distributed. In addition, the speed of the phragmoplast expansion during longitudinal cell divisions in stelar parenchymatic cells in the *ktn1-2* mutant was significantly slower compared to Col-0 (Figure S7; Videos S13, S14), corroborating previously published data (Komis et al., 2017).

## Discussion

The microtubule severing protein KATANIN1 plays a major role in the regulation of microtubule reorganizations in plant cells (Luptovčiak et al., 2017a). It is involved in both the regulation of cortical microtubule arrays (Burk et al., 2001; Stoppin-Mellet et al., 2006; Wightman and Turner, 2007; Nakamura et al., 2010; Lindeboom et al., 2013; Wightman et al., 2013) and of mitotic and cytokinetic microtubule arrays (Panteris et al., 2011, 2018; Panteris and Adamakis, 2012; Zhang et al., 2013; Komis et al., 2017). Therefore, microtubule organization underlying central developmental events of plant growth and morphogenesis is regulated by katanin-mediated severing activities. Regulation of mitotic, cytokinetic, and cortical microtubule arrays directly influences the progression of cell division, cell division plane orientation, but also post-mitotic cell expansion. Accordingly, *katanin1* mutants display dwarf phenotypes, influencing mainly root development [reviewed in Luptovčiak et al. (2017a)]. Appearance of abnormalities in the cell organization of the root apical meristem manifested through the observation of many oblique cross-walls and aberrant organization of cell files in *katanin1* mutants (Bichet et al., 2001; Webb et al., 2002; Panteris et al., 2011; Panteris and Adamakis, 2012; Komis et al., 2017) was thus anticipated as a consequence of disturbances in CDP orientation and anisotropic cell growth. Nevertheless, none of these studies employed a microscopic method allowing long-term observation of spatio-temporal organization of cell divisons in root apices leading to patterned formation of diverse tissues.

Here we analyzed patterns of cell divisions during the key developmental stages in growing primary roots and during LRP formation using advanced LSFM. This modern imaging method utilizes intact plants positioned in vertical orientation at the imaging stage of the microscope. Sustained plant growth and development is secured during the entire period of long-term imaging (Ovečka et al., 2015). Since excitation of fluorophores in imaged organs is restricted to the thin volume of light-sheet illumination, both phototoxicity and photobleaching are considerably reduced. Free rotation tool allowing multiangular imaging of sample is suitable for deep tissue and organ imaging (Ovečka et al., 2018). LSFM is thus providing sufficient spatial and temporal resolution to monitor topology and progress of cell divisions inside of living multicellular plant organs (Vyplelová et al., 2018). In *Arabidopsis thaliana*, multiscale LSFM imaging was used to study growth, patterning and development of primary root (Maizel et al., 2011; Sena et al., 2011; Costa et al., 2013; Ovečka et al., 2015; Novák et al., 2016, 2018) as well as different stages of lateral root formation (Lucas et al., 2013; Rosquete et al., 2013; Vermeer et al., 2014; von Wangenheim et al., 2016).

Such long-term LSFM live-cell imaging of dividing cells in growing roots of Col-0 and *ktn1-2* mutant plants carrying GFP-TUA6 microtulular marker provided an excellent tool to study the role of KATANIN1 in the establishment of root tissue patterning. We identified ectopic cell divisions in calyptrogen of *ktn1-2* mutant root, leading to malformation and disorganization of central columella cells in the root cap. Ectopic longitudinal cell divisions in the procambium of the main root resulted in the doubling of protoxylem forming cell files leading to the formation of ectopic, fully differentiated protoxylem vascular files within the entire root system. Another area where excessive ectopic longitudinal cell divisions were documented was the mature part of the root corresponding to the initiation of LRP formation. These cell divisions in the pericycle and stelar parenchyma of the central cylinder promoted radial multiplication of cells during LRP development, but also outside of the LRP formation zone. Frequencies of longitudinal cell divisions outside of the LRP formation zone were apparently higher in *ktn1-2* mutant.

Root growth in the examined *katanin1* mutants exhibits defects related to both disturbance of anisotropic cell growth and CDP orientation. As expected for KATANIN1 severing activity affecting organization of the cortical microtubule array, post-mitotically growing cells of the *fra2, lue1*, and *ktn1-2* show radial expansion resulting in a significant increase of the root width in post-meristematic zone. However, in the root meristem the most prominent defect was the abnormal cell organization. Incomplete cell walls were not observed, suggesting that *fra2, lue1*, and *ktn1-2* mutations do not affect the course of cell plate deposition during cytokinesis, which is consistent with similar phenotypic defects in *botero 1* and *ectopic root hair 3* mutants (Bichet et al., 2001; Webb et al., 2002; Panteris et al., 2011). Studies of mitotic microtubule arrays in the *fra2* and *lue1* mutants revealed the presence of multipolarly organized and frequently rotated mitotic spindles, which was suggested to be caused by the observed CDP misorientation in these mutants (Panteris et al., 2011). Using live cell imaging approach it was recently proved that KATANIN1 regulates dynamic properties of the interphase microtubules, but also the formation, maturation and positioning of the PPB, the mitotic spindle and the phragmoplast (Komis et al., 2017). It was found that defects in CDP orientation described in *katanin1* mutants are caused by delayed maturation, poor organization, or misorientation of PPB (Komis et al., 2017).

*KATANIN1* mutations also affected the development of lateral roots, but not LRP initiation, suggesting that pericycle cell specification is independent of KATANIN1 function during LRP establishment. However, in all three *katanin1* mutants the process of LRP formation and development were affected in a stage-independent manner, owing to uncoordinated oblique CDPs and ectopic cell divisions at early stages of LRP formation leading to disturbed cell patterning of emerging LRs. In a recent elegant study it was reported that another *katanin1* mutant, *botero*, showed drastically lower adventitious root formation in hypocotyls when treated with indole-3-butyric acid (Abu-Abied et al., 2015b). However, adventitious root formation follows a different developmental program than primary and lateral root development and strongly depends on hormone application (Verstraeten et al., 2014).

Plants of *katanin1* mutants displayed pleiotropic phenotypic defects during plant development, including lower fertility and seed set rate comparing to control plants (Luptovčiak et al., 2017b). Problems with fertility and seed set efficiency of *ktn1-2* mutant originated from abnormalities occuring during embryo development, encompassing abnormal cell division patterns in the embryo proper and in the hypophysis (Luptovčiak et al., 2017b). Defective patterning of the hypophysis pole of the developing embryo will necessarily affect post-embryonic root development. Such phenotypes of *katanin1* mutants can be partially explained by the absence of KATANIN1 functions related to the control of CDP orientation. However, the question that still remains is which KATANIN1-related mechanism is involved in the changing of pattern formation at different stages of root development through the promotion of ectopic longitudinal cell divisions. Although some important indirect effects may be involved, like KATANIN1 function in the regulation of microtubule bundling (Komis et al., 2017), this mechanism might involve regulation of cell division patterns between quiescent center, columella, and lateral root cap cells on one side, and proximal meristem on the other side. It might be realized through long-range signals including ROOT GROWTH FACTORS regulating PLETHORA gene family (Matsuzaki et al., 2010; Zhou et al., 2010) or ROS regulating cell division in the proximal meristem (Tsukagoshi et al., 2010; reviewed by Rahni et al., 2016). The hormonal regulation of the cytoskeleton rearrangements through KATANIN1 (e.g., Meier et al., 2001; Bouquin et al., 2003; Soga et al., 2010a,b) might represent an alternative mechanism controlling root cell division patterns. The impact of auxin on the orientation of the cell division plane has been proposed. This auxin-controlled mechanism is based on calcium-dependent regulation of katanin function, which supports either longitudinal or transversal alignment of microtubules as a key factor determining direction of cell division in early embryogenesis (reviewed by Winnicki, 2020). The *katanin1* mutant allele *botero1* was suggested to confer differential regulation of cytoskeletal genes, like *MAP65-3, MAP65-4*, and *TANGLED* among others (Abu-Abied et al., 2015a) intimately implicated in both the progression of cytokinesis (*MAP65-3*; Müller et al., 2004) and the regulation of cell division plane orientation (*MAP65-4*; Li et al., 2017; *TANGLED*; Walker et al., 2007). In this respect, differential proteomic analysis of Col-0, *fra2*, and *ktn1-2* mutants showed altered abundances of proteins involved in the microtubule and actin organization, as well as in hormonal regulation (Takáč et al., 2017). The explanation of the whole molecular mechanism, however, will require further studies.

KATANIN1-mediated microtubule severing was previously shown to be inherent to symmetry breaking in the cortical microtubule array either in the single cell or in the multicellular context (e.g., Uyttewaal et al., 2012; Lindeboom et al., 2013; Sassi et al., 2014). It was also found to lie behind the supracellular regulation of cell division plane orientation in a multicellular context (Jackson et al., 2019). KATANIN activity is thought to promote the self-organization of microtubules in response to many cues, including mechanical forces and hormones.

Cell division plane orientation is also related to cell geometry, when tensional forces within the cytoskeleton tends to force cell plate development along the shortest distance, minimizing the surface area of the cell plate [reffered as a Errera's rule, Besson and Dumais (2011)]. Similar to the organization of the cortical microtubule array, cell division plane orientation is additionally responsive to mechanical cues (Louveaux et al., 2016). The existing models have tackled successfully the distribution of cortical microtubules and determination of CDP orientation in epidermal tissues of shoot apical meristem or the development of multicellular glandular trichomes. However, it is not known how compressive or tensile forces are distributed within internal tissues of the root or other organs.

The shortest path of cell division plane orientation as formulated for epidermal tissues (Besson and Dumais, 2011) seems to apply to pericycle cells during the establishment of lateral root formation. This norm is not changing in the *katanin1* mutants examined herein, but rather extended, since we observed higher frequencies of longitudinal divisions. The doubling of protoxylem elements in *katanin1* mutants is also compliant to the same trend of increased longitudinal divisions in the procambium. In both cases cell divisions occur in embedded tissues, confined by the overlying cell files of the root; thus, it is likely that the mechanical environment of the internal root tissues of *katanin1* mutants is not much different than that of Col-0. The cells of root protoxylem in *katanin1* mutants are misshapped due to rearranged cortical microtubules and cell division plane reorientation, though they are able to develop normally and form fully developed protoxylem strands. Importantly, instead of single cell files, they are doubled. Mechanical cues and lateral forces inside the root tissue may apply to influence the developmental program to some extent, but most probably not only by CDP reorientation. Microtubules, and particularly KATANIN1-mediated microtubule reorganization, may be involved in specification of cell identity of the Arabidopsis root, which was shown for *ERH3* gene, encoding an Arabidopsis *KATANIN p60* homolog (Webb et al., 2002). Recent evidences based on the localization of extracellular leucine-rich repeats (LRR) receptor-like kinases (RLKs), named polarly localized kinases (PLKs), suggest that cell-to-cell communication provides essential cues for formative cell divisions (Campos et al., 2020). INFLORESCENCE AND ROOT APICES RECEPTOR KINASE (IRK) has been characterized as a transmembrane receptor kinase with polar localization in root cells that negatively regulates some specific cell divisions. This kinase perceives extrinsic cues likely from radially adjacent cells in the root and presents them intracellularly to control oriented (formative) cell divisions (Campos et al., 2020). Formative cell divisions thus comprise more complex mechanism, reflecting not only the changes in cytoskeleton reorganization and related changes in cell shape and mode of cell expansion. The overall data indicate that the pattern formation related to protoxylem development is rather affected by ectopic position of founder cells, originating from ectopic formative (periclinal) cell division.

According to previously published research, lateral root development can be triggered by external force application (e.g., through bending) and is responsive to auxin application as evidenced by increased amounts of the PIN1 auxin transporter in pericycle founder cells from which LRPs are originating [e.g., Omelyanchuk et al. (2016)]. Notably, induction of lateral root formation by mechanical bending is also associated with redistribution of PIN1 in protoxylem cells (Ditengou et al., 2008). In addition, the correct cell division plane orientation requires suppression of cytokinin signaling in pericycle founder cells (Moreira et al., 2013). This raises the possibility that the increased frequencies of longitudinal divisions in either the procambium or the pericycle of *ktn1-2* mutant might be indirectly evoked by molecular events controlling lateral root or vasculature development.

In summary, advanced long-term LSFM imaging of living plants at high spatio-temporal resolution revealed new interesting aspects of developmental pattern formation in roots. This study showed that ectopic cell divisions altered the cellular patterning and significantly contributed to the expanded root phenotypes of *A. thaliana katanin1* mutants.

## Data Availability Statement

The datasets generated for this study are available on request to corresponding authors.

## Author Contributions

MO performed the majority of the experiments presented hereby, characterized stem cell niche in cleared roots, documented lateral root formation and live cell imaging studies, and contributed with IL and OŠ to quantitative root phenotyping. GK documented root phenotypes by FM4-64. DS prepared transgenic line of *lue1* with microtubular marker. JŠ provided infrastructure and funding. MO, GK, and IL wrote the manuscript with input from co-authors. MO and JŠ conceived the study, designed the experiments, and supervised the work.

## Conflict of Interest

The authors declare that the research was conducted in the absence of any commercial or financial relationships that could be construed as a potential conflict of interest.

## Supplementary Material

Video S1 Z-projection of individual optical sections through primary root apex of Col-0 plant stably expressing a GFP-TUA6 microtubule marker.

Video S2 Z-projection of individual optical sections through primary root apex of *ktn1-2* mutant plant stably expressing a GFP-TUA6 microtubule marker.

Video S3 Z-projection of individual optical sections through elongation zone of the primary root apex of Col-0 plant stably expressing a GFP-TUA6 microtubule marker depicting cortical microtubules.

Video S4 Z-projection of individual optical sections through lateral root cap cell on the primary root apex of Col-0 plant stably expressing a GFP-TUA6 microtubule marker depicting cortical microtubules.

Video S5 Z-projection of individual optical sections through elongation zone of the primary root apex of *ktn1-2* plant stably expressing a GFP-TUA6 microtubule marker depicting cortical microtubules.

Video S6 Z-projection of individual optical sections through group of lateral root cap cells on the primary root apex of *ktn1-2* plant stably expressing a GFP-TUA6 microtubule marker depicting cortical microtubules.

Video S7 Growth of primary root of Col-0 plant stably expressing a GFP-TUA6 microtubule marker over the period of 3 h and 15 min stabilized to the y-axis.

Video S8 Growth of primary root of *ktn1-2* mutant plant stably expressing a GFP-TUA6 microtubule marker over the period of 3 h and 15 min stabilized to the y-axis.

Video S9 Longitudinal cell divisions in the central cylinder at the region of first lateral root primordium in Col-0 plant stably expressing a GFP-TUA6 microtubule marker over the period of 10 h.

Video S10 Maximum intensity projection and 3-D rendering of the central cylinder at the region of first lateral root primordium formation in Col-0 plant stably expressing a GFP-TUA6 microtubule marker recorded for the period of 10 h.

Video S11 Longitudinal cell divisions in the central cylinder at the region of first lateral root primordiumin *ktn1-2* mutant plant stably expressing a GFP-TUA6 microtubule marker over the period of 10 h.

Video S12 Maximum intensity projection and 3-D rendering of the central cylinder at the region of first lateral root primordium formation in *ktn1-2* mutant plant stably expressing a GFP-TUA6 microtubule marker recorded for the period of 10 h.

Video S13 Longitudinal cell division of one representative cell in the central cylinder at the region of first lateral root primordium formation in Col-0 plant stably expressing a GFP-TUA6 microtubule marker recorded for the period of 120 min.

Video S14 Longitudinal cell division of one representative cell in the central cylinder at the region of first lateral root primordium formation in *ktn1-2* mutant plant stably expressing a GFP-TUA6 microtubule marker recorded for the period of 130 min.

## References

[B1] Abu-Abied M. Mordehaev I. Sunil Kumar G. B. Ophir R. Wasteneys G. O. Sadot E. (2015a). Analysis of microtubule-associated-proteins during IBA-mediated adventitious root induction reveals KATANIN dependent and independent alterations of expression patterns. PLoS ONE 10:e0143828. 10.1371/journal.pone.0143828 26630265 PMC4668071

[B2] Abu-Abied M. RogovoyStelmakh O. Mordehaev I. Grumberg M. Elbaum R. Wasteneys G. O.. (2015b). Dissecting the contribution of microtubule behaviour in adventitious root induction. J. Exp. Bot. 66, 2813–2824. 10.1093/jxb/erv097 25788735 PMC4986881

[B3] Beck M. Komis G. Müller J. Menzel D. Šamaj J. (2010). Arabidopsis homologs of nucleus- and phragmoplast-localized kinase 2 and 3 and mitogen-activated protein kinase 4 are essential for microtubule organization. Plant Cell 22, 755–771. 10.1105/tpc.109.071746 20215588 PMC2861451

[B4] Beck M. Komis G. Ziemann A. Menzel D. Šamaj J. (2011). Mitogen-activated protein kinase 4 is involved in the regulation of mitotic and cytokinetic microtubule transitions in *Arabidopsis thaliana*. New Phytol. 189, 1069–1083. 10.1111/j.1469-8137.2010.03565.x 21155826

[B5] Besson S. Dumais J. (2011). Universal rule for the symmetric division of plant cells. Proc. Natl. Acad. Sci. U. S. A. 108, 6294–6299. 10.1073/pnas.1011866108 21383128 PMC3076879

[B6] Bichet A. Desnos T. Turner S. Grandjean O. Höfte H. (2001). BOTERO1 is required for normal orientation of cortical microtubules and anisotropic cell expansion in arabidopsis. Plant J. 25, 137–148. 10.1046/j.1365-313x.2001.00946.x 11169190

[B7] Bouquin T. Mattsson O. Naested H. Foster R. Mundy J. (2003). The arabidopsis lue1 mutant defines a katanin p60 ortholog involved in hormonal control of microtubule orientation during cell growth. J. Cell Sci.116, 791–801. 10.1242/jcs.00274 12571277

[B8] Burk D. H. Liu B. Zhong R. Morrison W. H. Ye Z. H. (2001). A katanin-like protein regulates normal cell wall biosynthesis and cell elongation. Plant Cell 13, 807–827. 10.2307/3871342 11283338 PMC135546

[B9] Burk D. H. Ye Z. H. (2002). Alteration of oriented deposition of cellulose microfibrils by mutation of a katanin-like microtubule-severing protein. Plant Cell 14, 2145–2160. 10.1105/tpc.003947 12215512 PMC150762

[B10] Buschmann H. Chan J. Sanchez-Pulido L. Andrade-Navarro M. A. Doonan J. H. Lloyd C. W. (2006). Microtubule-associated AIR9 recognizes the cortical division site at preprophase and cell-plate insertion. Curr. Biol. 16, 1938–1943. 10.1016/j.cub.2006.08.028 17027491

[B11] Camilleri C. Azimzadeh J. Pastuglia M. Bellini C. Grandjean O. Bouchez D. (2002). The arabidopsis TONNEAU2 gene encodes a putative novel protein phosphatase 2A regulatory subunit essential for the control of the cortical cytoskeleton. Plant Cell 14, 833–845. 10.1105/tpc.010402 11971138 PMC150685

[B12] Campos R. Goff J. Rodriguez-Furlan C. Van Norman J. M. (2020). The arabidopsis receptor kinase IRK is polarized and represses specific cell divisions in roots. Dev. Cell 52, 183–195. 10.1016/j.devcel.2019.12.001 31883775

[B13] Costa A. Candeo A. Fieramonti L. Valentini G. Bassi A. (2013). Calcium dynamics in root cells of *Arabidopsis thaliana* visualized with selective plane illumination microscopy. PLoS ONE 8:e75646. 10.1371/journal.pone.0075646 24146766 PMC3797704

[B14] Deinum E. E. Mulder B. M. (2013). Modelling the role of microtubules in plant cell morphology. Curr. Opin. Plant Biol. 16, 688–692. 10.1016/j.pbi.2013.10.001 24157061

[B15] Ditengou F. A. Teale W. D. Kochersperger P. Flittner K. A. Kneuper I. van der Graaff E.. (2008). Mechanical induction of lateral root initiation in *Arabidopsis thaliana*. Proc. Natl. Acad. Sci. U. S. A. 105, 18818–18823. 10.1073/pnas.0807814105 19033199 PMC2596224

[B16] Dolan L. Janmaat K. Willemsen V. Linstead P. Poethig S. Roberts K.. (1993). Cellular organisation of the *Arabidopsis thaliana* root. Development 119, 71–84. 8275865 10.1242/dev.119.1.71

[B17] Gutierrez R. Lindeboom J. J. Paredez A. R. Emons A. M. Ehrhardt D. W. (2009). Arabidopsis cortical microtubules position cellulose synthase delivery to the plasma membrane and interact with cellulose synthase trafficking compartments. Nat. Cell Biol. 11, 797–806. 10.1038/ncb1886 19525940

[B18] Hamada T. (2014). Microtubule organization and microtubule-associated proteins in plant cells. Int. Rev. Cell Mol. Biol. 312, 1–52. 10.1016/B978-0-12-800178-3.00001-4 25262237

[B19] Hartman J. J. Mahr J. McNally K. Okawa K. Iwamatsu A. Thomas S.. (1998). Katanin, a microtubule-severing protein, is a novel AAA ATPase that targets to the centrosome using a WD40-containing subunit. Cell 93, 277–287. 10.1016/S0092-8674(00)81578-0 9568719

[B20] Hartman J. J. Vale R. D. (1999). Microtubule disassembly by ATP-dependent oligomerization of the AAA enzyme katanin. Science 286, 782–785. 10.1126/science.286.5440.782 10531065

[B21] Jackson M. D. B. Duran-Nebreda S. Kierzkowski D. Strauss S. Xu H. Landrein B.. (2019). Global topological order emerges through local mechanical control of cell divisions in the arabidopsis shoot apical meristem. Cell Syst. 8, 53–65.e3. 10.1016/j.cels.2018.12.009 30660611 PMC6345583

[B22] Komis G. Luptovčiak I. Ovečka M. Samakovli D. Šamajová O. Šamaj J. (2017). Katanin effects on dynamics of cortical microtubules and mitotic arrays in *Arabidopsis thaliana* revealed by advanced live-cell imaging. Front. Plant Sci. 8:866. 10.3389/fpls.2017.00866 28596780 PMC5443160

[B23] Komis G. Mistrík M. Šamajová O. Doskočilová A. Ovečka M. Illés P.. (2014). Dynamics and organization of cortical microtubules as revealed by superresolution structured illumination microscopy. Plant Physiol. 165, 129–148. 10.1104/pp.114.238477 24686112 PMC4012574

[B24] Kosetsu K. Matsunaga S. Nakagami H. Colcombet J. Sasabe M. Soyano T.. (2010). The MAP kinase MPK4 is required for cytokinesis in *Arabidopsis thaliana*. Plant Cell 22, 3778–3790. 10.1105/tpc.110.077164 21098735 PMC3015120

[B25] Li H. Sun B. Sasabe M. Deng X. Machida Y. Lin H.. (2017). Arabidopsis MAP65-4 plays a role in phragmoplast microtubule organization and marks the cortical cell division site. New Phytol. 215, 187–201. 10.1111/nph.14532 28370001

[B26] Lindeboom J. J. Nakamura M. Hibbel A. Shundyak K. Gutierrez R. Ketelaar T.. (2013). A mechanism for reorientation of cortical microtubule arrays driven by microtubule severing. Science 342:1245533. 10.1126/science.1245533 24200811

[B27] Livanos P. Müller S. (2019). Division plane establishment and cytokinesis. Annu. Rev. Plant Biol. 70, 239–267. 10.1146/annurev-arplant-050718-100444 30795703

[B28] Louveaux M. Julien J. D. Mirabet V. Boudaoud A. Hamant O. (2016). Cell division plane orientation based on tensile stress in *Arabidopsis thaliana*. Proc. Natl. Acad. Sci. U. S. A. 113, E4294–E4303. 10.1073/pnas.1600677113 27436908 PMC4968720

[B29] Lucas M. Kenobi K. von Wangenheim D. Voβ U. Swarup K. De Smet I.. (2013). Lateral root morphogenesis is dependent on the mechanical properties of the overlaying tissues. Proc. Natl. Acad. Sci. U. S. A. 110, 5229–5234. 10.1073/pnas.1210807110 23479644 PMC3612681

[B30] Luptovčiak I. Komis G. Takáč T. Ovečka M. Šamaj J. (2017a). Katanin: a sword cutting microtubules for cellular, developmental, and physiological purposes. Front. Plant Sci. 8:1982. 10.3389/fpls.2017.01982 29209346 PMC5702333

[B31] Luptovčiak I. Samakovli D. Komis G. Šamaj J. (2017b). KATANIN1 is essential for embryogenesis and seed formation in Arabidopsis. Front. Plant Sci. 8:728. 10.3389/fpls.2017.00728 28529520 PMC5418335

[B32] Maizel A. von Wangenheim D. Federici F. Haseloff J. Stelzer E. H. (2011). High-resolution live imaging of plant growth in near physiological bright conditions using light sheet fluorescence microscopy. Plant J. 68, 377–385. 10.1111/j.1365-313X.2011.04692.x 21711399

[B33] Malamy J. E. Benfey P. N. (1997). Organization and cell differentiation in lateral roots of *Arabidopsis thaliana*. Development 124, 33–44. 9006065 10.1242/dev.124.1.33

[B34] Matsuzaki Y. Ogawa-Ohnishi M. Mori A. Matsubayashi Y. (2010). Secreted peptide signals required for maintenance of root stem cell niche in arabidopsis. Science 329, 1065–1067. 10.1126/science.1191132 20798316

[B35] Meier C. Bouquin T. Nielsen M. E. Raventos D. Mattsson O. Rocher A.. (2001). Gibberellin response mutants identified by luciferase imaging. Plant J. 25, 509–519. 10.1046/j.1365-313x.2001.00980.x 11309141

[B36] Metzinger C. A. Bergmann D. C. (2010). Plant asymmetric cell division regulators: pinch-hitting for PARs? F1000 Biol. Rep. 2:25. 10.3410/B2-25 20948808 PMC2948360

[B37] Moreira S. Bishopp A. Carvalho H. Campilho A. (2013). AHP6 inhibits cytokinin signaling to regulate the orientation of pericycle cell division during lateral root initiation. PLoS ONE 8:e56370. 10.1371/journal.pone.0056370 23457561 PMC3572949

[B38] Müller J. Beck M. Mettbach U. Komis G. Hause G. Menzel D.. (2010). Arabidopsis MPK6 is involved in cell division plane control during early root development, and localizes to the pre-prophase band, phragmoplast, trans-Golgi network and plasma membrane. Plant J. 61, 234–248. 10.1111/j.1365-313X.2009.04046.x 19832943

[B39] Müller S. (2012). Universal rules for division plane selection in plants. Protoplasma 249, 239–253. 10.1007/s00709-011-0289-y 21611883

[B40] Müller S. Han S. Smith L. G. (2006). Two kinesins are involved in the spatial control of cytokinesis in *Arabidopsis thaliana*. Curr. Biol. 16, 888–894. 10.1016/j.cub.2006.03.034 16682350

[B41] Müller S. Smertenko A. Wagner V. Heinrich M. Hussey P. J. Hauser M. T. (2004). The plant microtubule-associated protein AtMAP65-3/PLE is essential for cytokinetic phragmoplast function. Curr. Biol. 14, 412–417. 10.1016/j.cub.2004.02.032 15028217 PMC2867789

[B42] Nakamura M. (2015). Microtubule nucleating and severing enzymes for modifying microtubule array organization and cell morphogenesis in response to environmental cues. New Phytol. 205, 1022–1027. 10.1111/nph.12932 25729799

[B43] Nakamura M. Ehrhardt D. W. Hashimoto T. (2010). Microtubule and katanin-dependent dynamics of microtubule nucleation complexes in the acentrosomal arabidopsis cortical array. Nat. Cell Biol.12, 1064–1070. 10.1038/ncb2110 20935636

[B44] Novák D. Kuchařová A. Ovečka M. Komis G. Šamaj J. (2016). Developmental nuclear localization and quantification of GFP-tagged EB1c in arabidopsis root using light-sheet microscopy. Front. Plant Sci. 6:1187. 10.3389/fpls.2015.01187 26779221 PMC4700127

[B45] Novák D. Vadovič P. Ovečka M. Šamajová O. Komis G. Colcombet J.. (2018). Gene expression pattern and protein localization of arabidopsis phospholipase D alpha 1 revealed by advanced light-sheet and super-resolution microscopy. Front. Plant Sci. 9:371. 10.3389/fpls.2018.00371 29628934 PMC5877115

[B46] Omelyanchuk N. A. Kovrizhnykh V. V. Oshchepkova E. A. Pasternak T. Palme K. Mironova V. V. (2016). A detailed expression map of the PIN1 auxin transporter in *Arabidopsis thaliana* root. BMC Plant Biol. 16:5. 10.1186/s12870-015-0685-0 26821586 PMC4895256

[B47] Ovečka M. Vaškebová L. Komis G. Luptovčiak I. Smertenko A. Šamaj J. (2015). Preparation of plants for developmental and cellular imaging by light-sheet microscopy. Nat. Protoc. 10, 1234–1247. 10.1038/nprot.2015.081 26203821

[B48] Ovečka M. von Wangenheim D. Tomančák P. Šamajová O. Komis G Šamaj J. (2018). Multiscale imaging of plant development by light-sheet fluorescence microscopy. Nat. Plants 4, 639–650. 10.1038/s41477-018-0238-2 30185982

[B49] Panteris E. Adamakis I. D. (2012). Aberrant microtubule organization in dividing root cells of p60-katanin mutants. Plant Sig. Behav. 7, 16–18. 10.4161/psb.7.1.18358 22301959 PMC3357358

[B50] Panteris E. Adamakis I. D. Voulgari G. Papadopoulou G. (2011). A role for katanin in plant cell division: microtubule organization in dividing root cells of fra2 and lue1*Arabidopsis thaliana* mutants. Cytoskeleton 68, 401–413. 10.1002/cm.20522 21721142

[B51] Panteris E. Diannelidis B. E. Adamakis I. S. (2018). Cortical microtubule orientation in *Arabidopsis thaliana* root meristematic zone depends on cell division and requires severing by katanin. J. Biol. Res-Thessalon. 25:12. 10.1186/s40709-018-0082-6 29942798 PMC6002977

[B52] Parizot B. Laplaze L. Ricaud L. Boucheron-Dubuisson E. Bayle V. Bonke M.. (2008). Diarch symmetry of the vascular bundle in arabidopsis root encompasses the pericycle and is reflected in distich lateral root initiation. Plant Physiol. 146, 140–148. 10.1104/pp.107.107870 17993548 PMC2230548

[B53] Polko J. K. Kieber J. J. (2019). The regulation of cellulose biosynthesis in plants. Plant Cell 31, 282–296. 10.1105/tpc.18.00760 30647077 PMC6447023

[B54] Rahni R. Efroni I. Birnbaum K. D. (2016). A case for distributed control of local stem cell behavior in plants. Dev. Cell 38, 635–642. 10.1016/j.devcel.2016.08.015 27676436 PMC5076558

[B55] Rasmussen C. G. Humphries J. A. Smith L. G. (2011). Determination of symmetric and asymmetric division planes in plant cells. Annu. Rev. Plant Biol. 62, 387–409. 10.1146/annurev-arplant-042110-103802 21391814

[B56] Rosquete M. R. von Wangenheim D. Marhavý P. Barbez E. Stelzer E. H. Benková E.. (2013). An auxin transport mechanism restricts positive orthogravitropism in lateral roots. Curr. Biol. 23, 817–822. 10.1016/j.cub.2013.03.064 23583551

[B57] Šamajová O. Komis G. Šamaj J. (2014). Immunofluorescent localization of MAPKs and colocalization with microtubules in arabidopsis seedling whole-mount probes. Methods Mol. Biol. 1171, 107–115. 10.1007/978-1-4939-0922-3_9 24908123

[B58] Sampathkumar A. Lindeboom J. J. Debolt S. Gutierrez R. Ehrhardt D. W. Ketelaar T.. (2011). Live cell imaging reveals structural associations between the actin and microtubule cytoskeleton in arabidopsis. Plant Cell 23, 2302–2313. 10.1105/tpc.111.087940 21693695 PMC3160026

[B59] Sasaki T. Tsutsumi M. Otomo K. Murata T. Yagi N. Nakamura M.. (2019). Novel katanin-tethering machinery accelerates cytokinesis. Curr. Biol. 29, 4060–4070.e3. 10.1016/j.cub.2019.09.049 31735673

[B60] Sassi M. Ali O. Boudon F. Cloarec G. Abad U. Cellier C.. (2014). An auxin-mediated shift toward growth isotropy promotes organ formation at the shoot meristem in Arabidopsis. Curr. Biol. 24, 2335–2342. 10.1016/j.cub.2014.08.036 25264254

[B61] Sena G. Frentz Z. Birnbaum K. D. Leibler S. (2011). Quantitation of cellular dynamics in growing arabidopsis roots with light sheet microscopy. PLoS ONE 6:e21303. 10.1371/journal.pone.0021303 21731697 PMC3120859

[B62] Smékalová V. Luptovčiak I. Komis G. Šamajová O. Ovečka M. Doskočilová A.. (2014). Involvement of YODA and mitogen activated protein kinase 6 in arabidopsis post-embryogenic root development through auxin up-regulation and cell division plane orientation. New Phytol. 203, 1175–1193. 10.1111/nph.12880 24923680 PMC4414326

[B63] Smertenko A. Assaad F. Baluška F. Bezanilla M. Buschmann H. Drakakaki G.. (2017). Plant cytokinesis: terminology for structures and processes. Trends Cell Biol. 27, 885–894. 10.1016/j.tcb.2017.08.008 28943203

[B64] Soga K. Yamaguchi A. Kotake T. Wakabayashi K. Hoson T. (2010a). 1-aminocyclopropane-1-carboxylic acid ACC-induced reorientation of cortical microtubules is accompanied by a transient increase in the transcript levels of gamma-tubulin complex and katanin genes in azuki bean epicotyls. J. Plant Physiol. 167, 1165–1171. 10.1016/j.jplph.2010.04.001 20451287

[B65] Soga K. Yamaguchi A. Kotake T. Wakabayashi K. Hoson T. (2010b). Transient increase in the levels of g-tubulin complex and katanin are responsible for reorientation by ethylene and hypergravity of cortical microtubules. Plant Signal. Behav. 5, 1480–1482. 10.4161/psb.5.11.13561 21051953 PMC3115261

[B66] Spinner L. Gadeyne A. Belcram K. Goussot M. Moison M. Duroc Y.. (2013). A protein phosphatase 2A complex spatially controls plant cell division. Nat. Commun. 4:1863. 10.1038/ncomms2831 23673648

[B67] Stoppin-Mellet V. Gaillard J. Timmers T. Neumann E. Conway J. Vantard M. (2007). Arabidopsis katanin binds microtubules using a multimeric microtubule-binding domain. Plant Physiol. Bioch. 45, 867–877. 10.1016/j.plaphy.2007.09.005 17977001

[B68] Stoppin-Mellet V. Gaillard J. Vantard M. (2006). Katanin's severing activity favors bundling of cortical microtubules in plants. Plant J. 46, 1009–1017. 10.1111/j.1365-313X.2006.02761.x 16805733

[B69] Takáč T. Šamajová O. Luptovčiak I. Pechan T. Šamaj J. (2017). Feedback microtubule control and microtubule-actin cross-talk in arabidopsis revealed by integrative proteomic and cell biology analysis of KATANIN1 mutants. Mol. Cell. Proteomics 16, 1591–1609. 10.1074/mcp.M117.068015 28706004 PMC5587860

[B70] Tsukagoshi H. Busch W. Benfey P. N. (2010). Transcriptional regulation of ROS controls transition from proliferation to differentiation in the root. Cell 143, 606–616. 10.1016/j.cell.2010.10.020 21074051

[B71] Uyttewaal M. Burian A. Alim K. Landrein B. Borowska-Wykret D. Dedieu A.. (2012). Mechanical stress acts via katanin to amplify differences in growth rate between adjacent cells in Arabidopsis. Cell 149, 439–451. 10.1016/j.cell.2012.02.048 22500806

[B72] Van Norman J. M. (2016). Asymmetry and cell polarity in root development. Dev. Biol. 17, 165–174. 10.1016/j.ydbio.2016.07.009 27426272

[B73] Vavrdová T. Šamajová O. Křenek P. Ovečka M. Floková P. Šnaurová R.. (2019). Multicolour three dimensional structured illumination microscopy of immunolabeled plant microtubules and associated proteins. Plant Methods 15:22. 10.1186/s13007-019-0406-z 30899319 PMC6408805

[B74] Vermeer J. E. von Wangenheim D. Barberon M. Lee Y. Stelzer E. H. Maizel A.. (2014). A spatial accommodation by neighboring cells is required for organ initiation in arabidopsis. Science 343, 178–183. 10.1126/science.1245871 24408432

[B75] Verstraeten I. Schotte S. Geelen D. (2014). Hypocotyl adventitious root organogenesis differs from lateral root development. Front. Plant Sci. 5:495. 10.3389/fpls.2014.00495 25324849 PMC4179338

[B76] von Wangenheim D. Fangerau J. Schmitz A. Smith R. S. Leitte H. Stelzer E. H.. (2016). Rules and self-organizing properties of postembryonic plant organ cell division patterns. Curr. Biol. 26, 439–449. 10.1016/j.cub.2015.12.047 26832441

[B77] Vyplelová P. Ovečka M. Komis G. Šamaj J. (2018). Advanced microscopy methods for bioimaging of mitotic microtubules in plants. Method. Cell Biol. 145, 129–158. 10.1016/bs.mcb.2018.03.019 29957201

[B78] Walker K. L. Müller S. Moss D. Ehrhardt D. W. Smith L. G. (2007). Arabidopsis TANGLED identifies the division plane throughout mitosis and cytokinesis. Curr. Biol. 17, 1827–1836. 10.1016/j.cub.2007.09.063 17964159 PMC2134921

[B79] Wang C. Liu W. Wang G. Li J. Dong L. Han L.. (2017). KTN80 confers precision to microtubule severing by specific targeting of katanin complexes in plant cells. EMBO J. 36, 3435–3447. 10.15252/embj.201796823 28978669 PMC5709764

[B80] Webb M. Jouannic S. Foreman J. Linstead P. Dolan L. (2002). Cell specification in the arabidopsis root epidermis requires the activity of ECTOPIC ROOT HAIR 3-a katanin-p60 protein. Development 129, 123–131. 11782406 10.1242/dev.129.1.123

[B81] Wightman R. Chomicki G. Kumar M. Carr P. Turner S. R. (2013). SPIRAL2 determines plant microtubule organization by modulating microtubule severing. Curr. Biol. 23, 1902–1907. 10.1016/j.cub.2013.07.061 24055158 PMC3793865

[B82] Wightman R. Turner S. R. (2007). Severing at sites of microtubule crossover contributes to microtubule alignment in cortical arrays. Plant J. 52, 742–751. 10.1111/j.1365-313X.2007.03271.x 17877711

[B83] Winnicki K. (2020). The winner takes it all: auxin–the main player during plant embryogenesis. Cells 9:606. 10.3390/cells9030606 32138372 PMC7140527

[B84] Xu X. M. Zhao Q. Rodrigo-Peiris T. Brkljacic J. He C. S. Müller S.. (2008). RanGAP1 is a continuous marker of the arabidopsis cell division plane. Proc. Natl. Acad. Sci. U. S. A. 105, 18637–18642. 10.1073/pnas.0806157105 19011093 PMC2587610

[B85] Zhang Q. Fischel E. Bertoche T. Dixit R. (2013). Microtubule severing at crossover sites by katanin generates order cortical microtubular arrays in arabidopsis. Curr. Biol. 23, 2191–2195. 10.1016/j.cub.2013.09.018 24206847

[B86] Zhou W. Wei L. Xu J. Zhai Q. Jiang H. Chen R.. (2010). Arabidopsis tyrosylprotein sulfotransferase acts in the auxin/PLETHORA pathway in regulating postembryonic maintenance of the root stem cell niche. Plant Cell 22, 3692–3709. 10.1105/tpc.110.075721 21045165 PMC3015123

