# Supplementary material for: Spatiotemporal Pattern of Ectopic Cell Divisions Contribute to Mis-Shaped Phenotype of Primary and Lateral Roots of katanin1 Mutant
Source: Front Plant Sci. 2020 Jun 9;11:734. doi: 10.3389/fpls.2020.00734 (PMC7296145; doi:10.3389/fpls.2020.00734)
Supplement: Supplementary file 1 [file Data_Sheet_1.pdf]

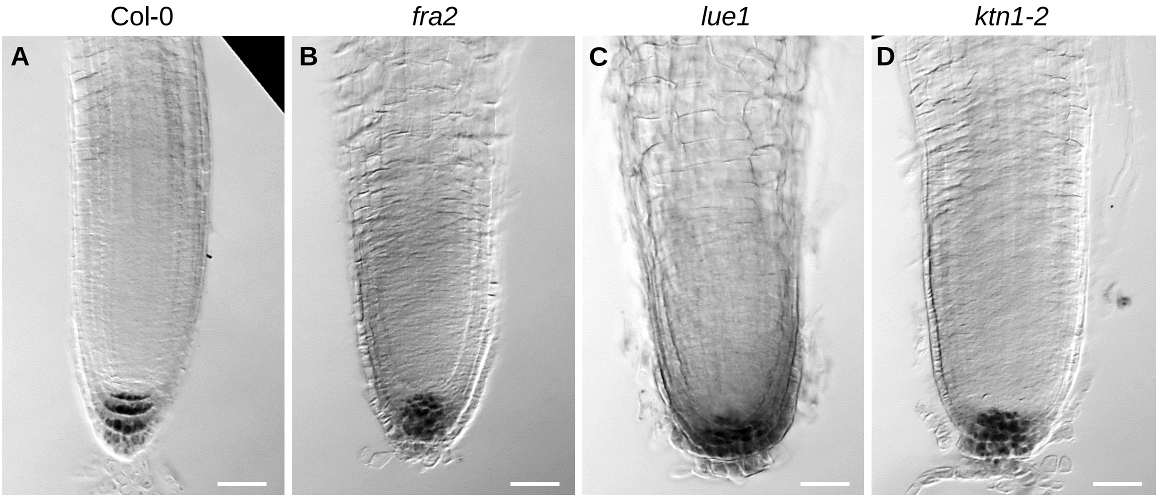

Figure S1. Overview of root tip anatomy of *katanin1* mutants after fixation, clearing and staining with Lugol solution. (**A-D**) Structure of root cap and distribution of starch grains in columella cells in Col-0 (**A**), *fra2* mutant (**B**), *lue1* mutant (**C**) and *ktn1-2* mutant (**D**). Bar: (**A-D**) 500 µm.

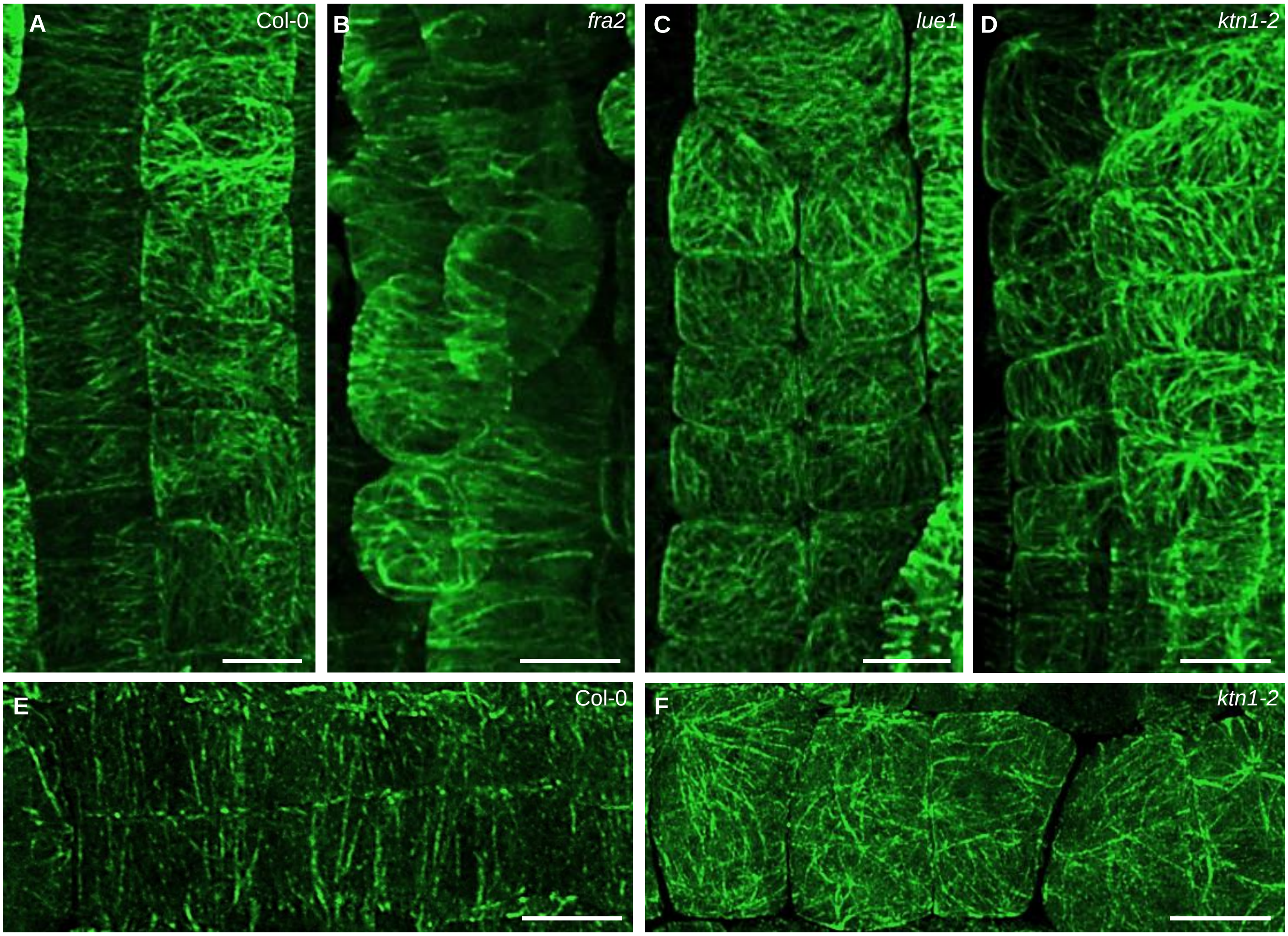

Figure S2. Whole mount immunofluorescence localization of cortical microtubules in root epidermal cells of *Arabidopsis* wild type Col-0 and *katanin1* mutants. (**A-D**) Group of root epidermal cells showing predominantly transverse and parallel organization of cortical microtubules in wild type Col-0 (**A**), less ordered transverse pattern of orientation in *fra2* (**B**) and more random non-regular orientations in *lue1* (**C**) and *ktn1-2* (**D**) mutants. (**E-F**) Cortical microtubules transversely oriented in wild type Col-0 (**E**) and randomly oriented in *ktn1-2* mutant (**F**) with respect to the root axis (in left-to-right direction). Microtubules in fixed samples were immunolocalized using anti-α tubulin primary and Alexa Fluor 488-conjugated secondary antibodies. Bar: (**A-D**) 10 μm; (**E-F**) 5 μm.

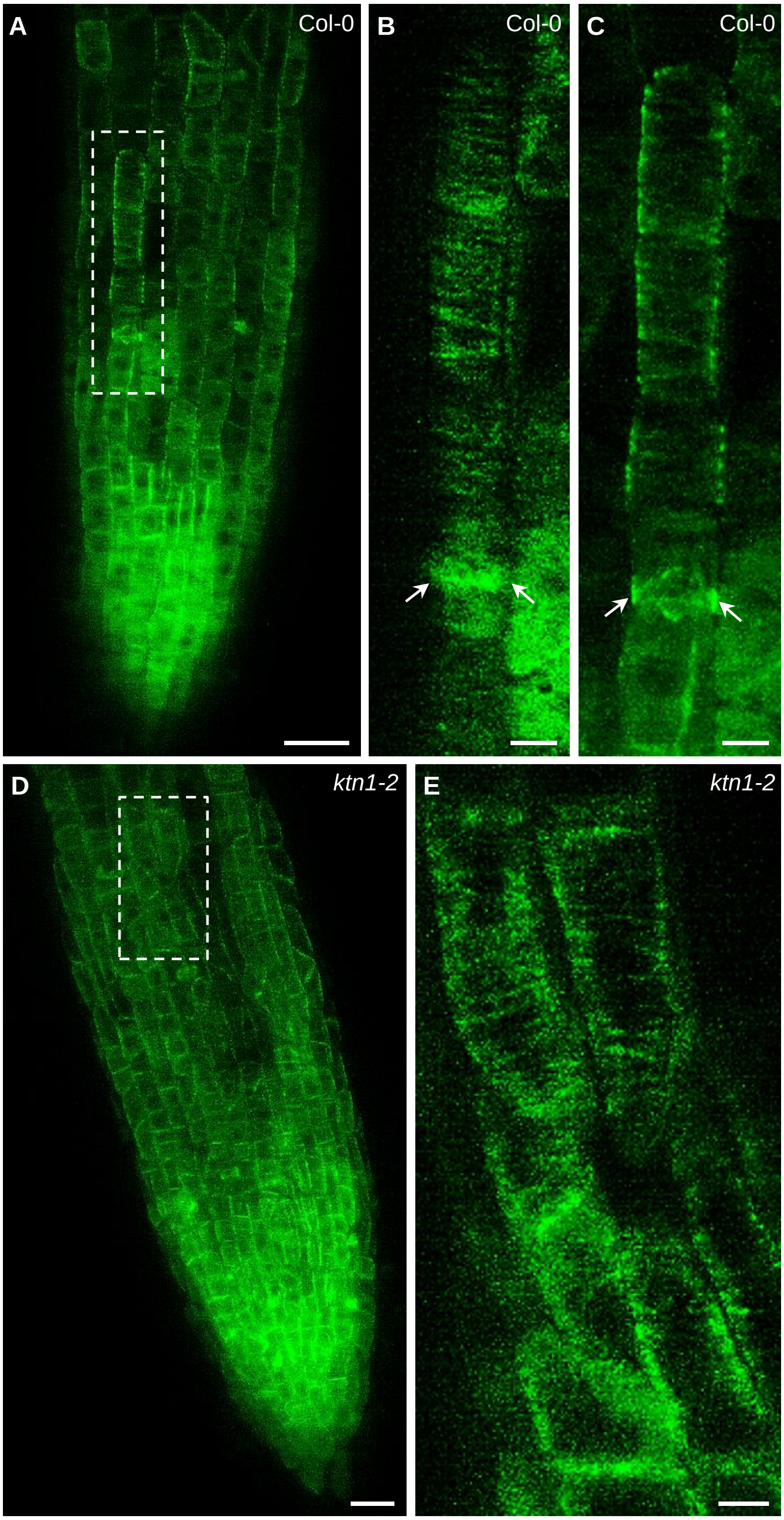

Figure S3. Visualization of epidermis and lateral root cap in primary root of wild type Col-0 and *ktn1-2* mutant plants stably carrying a GFP-TUA6 microtubule marker using light-sheet microscopy. (**A-C**) Microtubules in surface root cell layers of Col-0. Boxed area in (**A**) depicts lateral root cap cells showing cortical microtubules and PPB (arrows) in superficial (**B**) and median (**C**) optical sections. (**D-E**) Microtubules in surface root cell layers of *ktn1-2* mutant. Boxed area in (**D**) depicts lateral root cap cells showing cortical microtubules from individual optical sections (**E**). Bar: (**A,D**) 20 μm; (**B,C,E**) 5 μm.

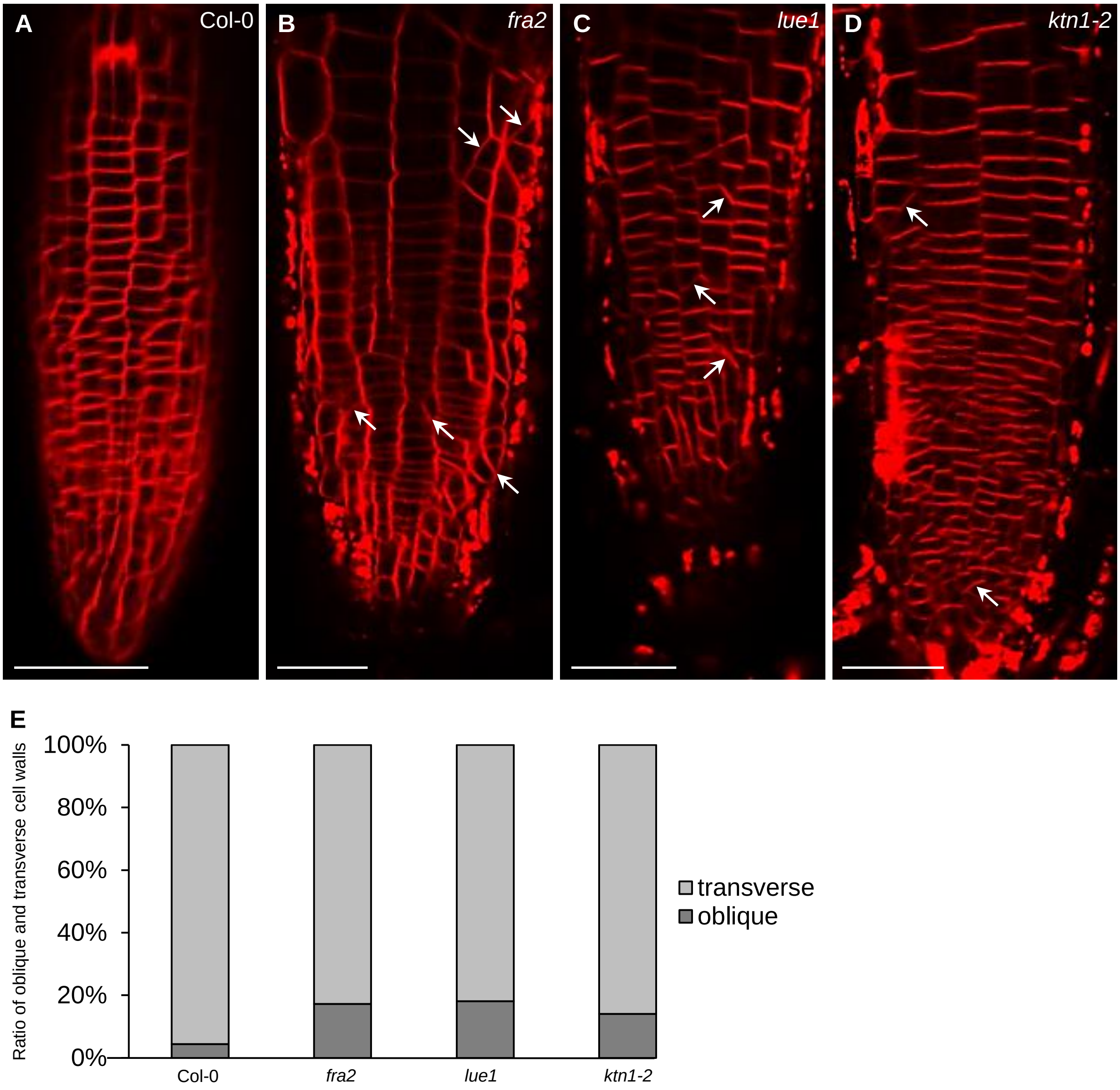

Figure S4. Cell division plane orientation in root epidermis of Col-0 and *katanin1* mutants stained with FM4-64. (**A**) Root epidermal cells of Col-0 seedlings showing a regular patterning. (**B-D**) Appearance of oblique cell divisions (arrows) in the root epidermis of *fra2* mutant (**B**), *lue1* mutant (**C**) and *ktn1-2* mutant (**D**) primary root. (**E**) Quantitative evaluation of oblique cell wall occurrence in epidermal cells of primary roots of Col-0 (n = 1273 cell walls), *fra2* (n = 1330 cell walls), *lue1* (n = 1321 cell walls) and *ktn1-2* (n = 1396 cell walls) mutants. Bars: (**A-D**) 50 μm.

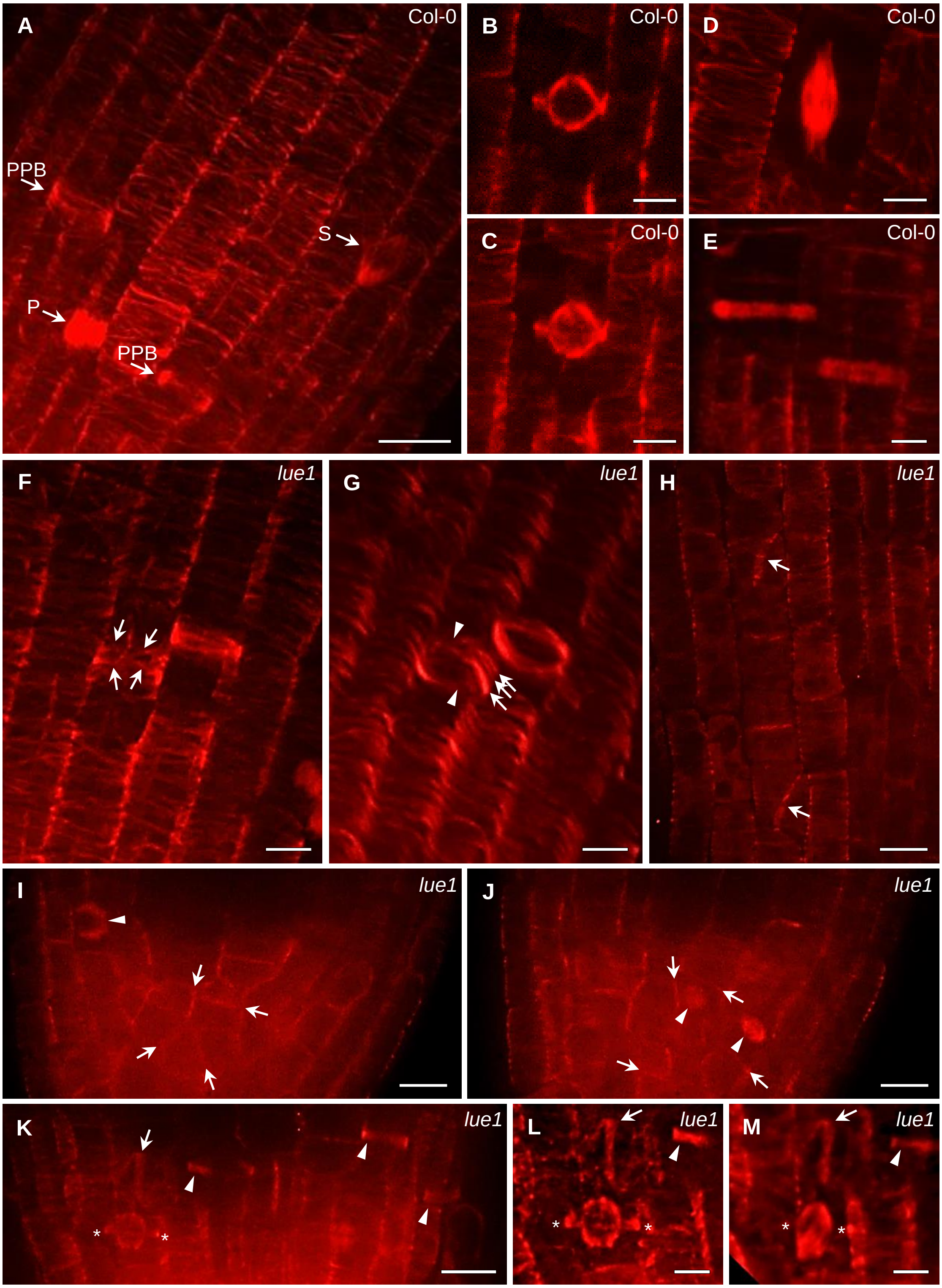

Figure S5. Structural organization of microtubules in cells of the root apex in *lue1* mutants stably expressing a cauliflower mosaic virus 35S promoter–driven mCherry fusion of the TUA5 (*35S::mCherry:TUA5*). (**A-E**) Expression of mCherry labelled TUA5 in transgenic Col-0 plants showing control root phenotype. Overview of microtubules in dividing and non-dividing root meristematic cells. Highlighted are microtubules of PPB (**A-C**), mitotic spindle (S; **A,D**) and phragmoplast (P; **A,E**) in dividing cells. Note the perpendicular orientation of PPB and phragmoplasts, and parallel orientation of mitotic spindles to the longitudinal root axis. (**F-M**) Expression of mCherry labelled TUA5 in transgenic *lue1* plants with short root phenotype. (**F,G**) Disorganized arrangement and orientation of microtubules in PPB in transgenic *lue1* plants. Microtubules had loosened organization and non-parallel mirotubules were present (arrows; **F**). The structure of PPB was not complete (arrowheads; **G**) and it contained several individual rings (arrows; **G**). (**H**) Presence of oblique and shifted cross cell walls. (**I,J**) Microtubules in central columella cells of the root cap in transgenic *lue1* plants. Positions of abnormally shifted cell division planes are indicated by arrows (**I,J**). Arrowheads indicate phragmoplast in anticlinal cell division (**I**) and shifted mitotic spindles (**J**). (**K-M**) Presence of tilted and abnormally positioned PPB in meristematic cell (arrow) in transgenic *lue1* plants. Arrowheads indicate normally oriented PPBs (**K-M**). Asterisks indicate progression of the cell division from early prophase (**K,L**) to metaphase (**M**). Bars: (**B-E,L,M**) 5 µm; (**A,F-K**) 10 µm.

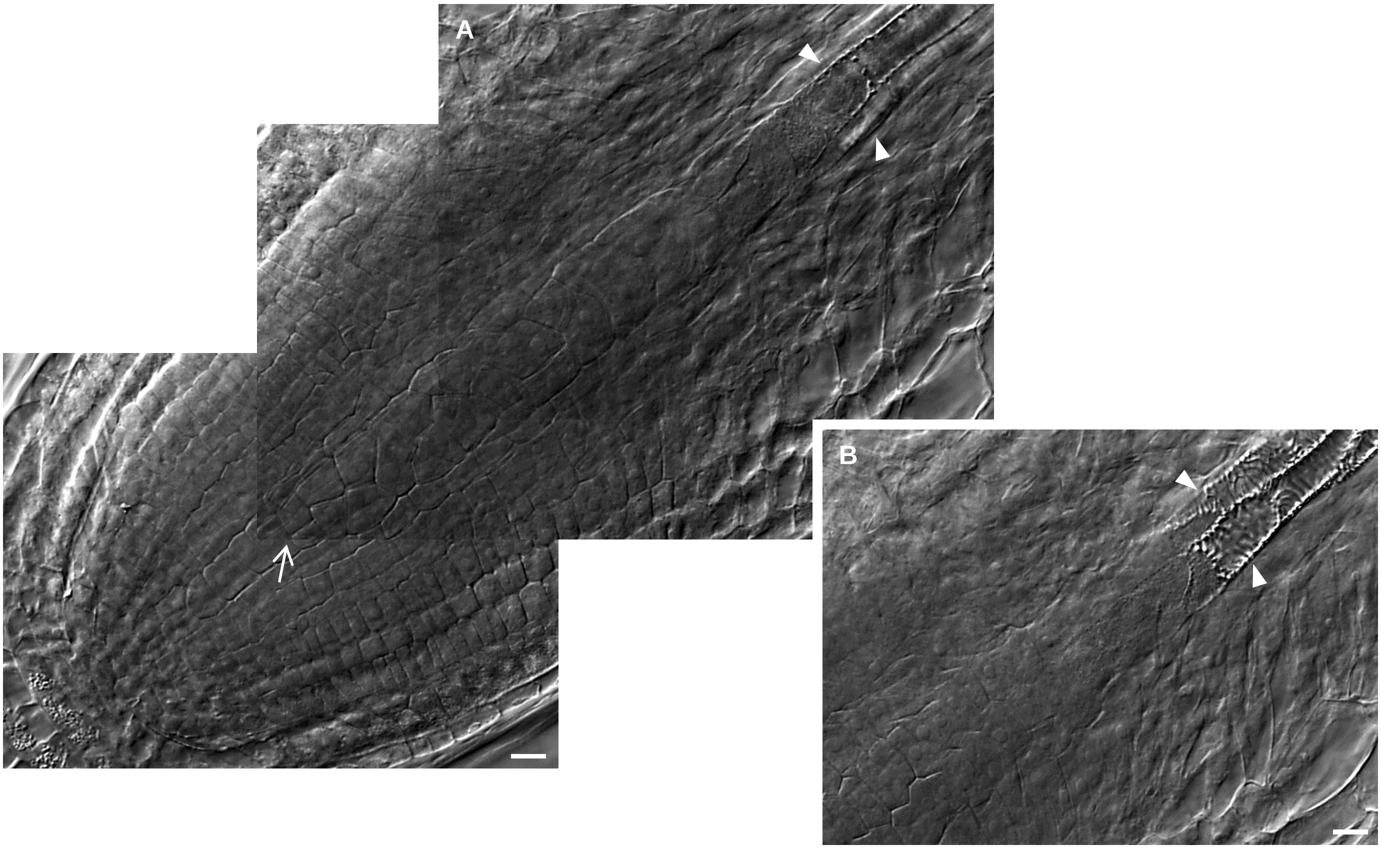

Figure S6. Cell lineage of procambium cell file and differentiation of protoxylem files in the apex of primary root of *ktn1-2* mutant. Sample from the clearing preparation showing duplication of protoxylem files (**A,B**, arrowheads). Cell file in the procambium contains cells with abnormal size, shape and position. Abnormal ectopic T-shaped longitudinal cell division is indicated by arrow (**A**). Bars: 10 μm.

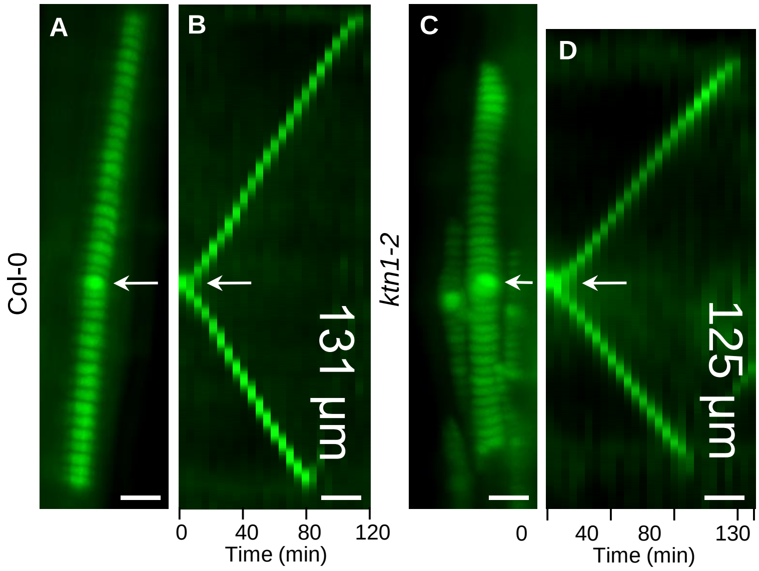

Figure S7. Progression of one representative longitudinal cell division in the central cylinder at the region of first lateral root primordium formation in Col-0 and *ktn1-2* mutant plants stably expressing a GFP-TUA6 microtubule marker. Speed of the cell divisions in Col-0 (**A**) and *ktn1-2* mutant (**C**) is visualized as time lapse maximum intensity projections (**A,C**) and depicted from kymographs (**B,D**). Arrows point to the place of early phragmoplast initiation (**A-D**). Distances of the margins of late phragmoplasts are indicated in pictures in µm (**B,D**). Bars: (**A-D**) 10 µm.
